# Tuning the optical and magnetic properties of Tb^3+^-doped ZnO quantum dots: structural investigation and Judd–Ofelt theoretical analysis

**DOI:** 10.1039/d6na00383d

**Published:** 2026-07-21

**Authors:** N. T. Hien, N. T. M. Thuy, N. T. K. Van, N. T. Luyen, L. V. Hoang, V. H. Yen, T. Ngoc, L. X. Hung, N. X. Ca

**Affiliations:** a Institute of Science and Technology, TNU-University of Sciences Thai Nguyen Vietnam nguyenxuanca@tnus.edu.vn; b Faculty of Physics, TNU-University of Education Thai Nguyen Vietnam; c Faculty of Engineering and Technology, TNU – University of Information and Communication Technology Thai Nguyen Vietnam; d Institute of Research and Development, Duy Tan University Da Nang 550000 Vietnam; e Faculty of Environment and Natural Sciences, Duy Tan University Da Nang 550000 Vietnam; f Faculty of Basic Sciences, Thai Binh University of Medicine and Pharmacy Hung Yen Vietnam

## Abstract

Tb^3+^-doped ZnO quantum dots (ZnO:Tb^3+^ QDs) with Tb^3+^ concentrations of 0–7 mol% were synthesized by a wet-chemical precipitation method to tune their optical and magnetic properties. XRD and Rietveld refinement confirmed the formation of hexagonal wurtzite ZnO without detectable secondary phases, while lattice expansion and increased microstrain suggested the incorporation of Tb^3+^ ions into the ZnO matrix. XPS and EDX mapping verified the presence and homogeneous distribution of Tb species. UV-vis absorption spectra showed a red shift of the absorption edge and bandgap narrowing with increasing Tb^3+^ content, indicating enhanced defect-related disorder. The PL spectra exhibited characteristic Tb^3+^ emissions, dominated by the green ^5^D_4_ → ^7^F_5_ transition at approximately 546 nm. The emission intensity increased up to 5 mol% Tb^3+^ and then decreased due to concentration quenching. Judd–Ofelt analysis revealed enhanced local asymmetry and covalent character around Tb^3+^ ions, with ZnO:Tb5% showing favorable radiative parameters for green emission. Lifetime analysis confirmed increased non-radiative relaxation at higher Tb^3+^ concentrations. Magnetic measurements showed that pristine ZnO QDs were diamagnetic, whereas Tb^3+^ doping induced weak room-temperature ferromagnetic-like behavior, with the highest saturation magnetization obtained for ZnO:Tb3%. These results demonstrate that Tb^3+^ doping is an effective strategy for simultaneously tailoring the luminescent and magnetic functionalities of ZnO QDs for photonic, magneto-optical, and spintronic applications.

## Abstract 2

Tb^3+^-doped ZnO quantum dots (ZnO:Tb^3+^ QDs) with Tb^3+^ concentrations of 0–7 mol% were synthesized by a wet-chemical precipitation method to tune their optical and magnetic properties.

## Graphical abstract

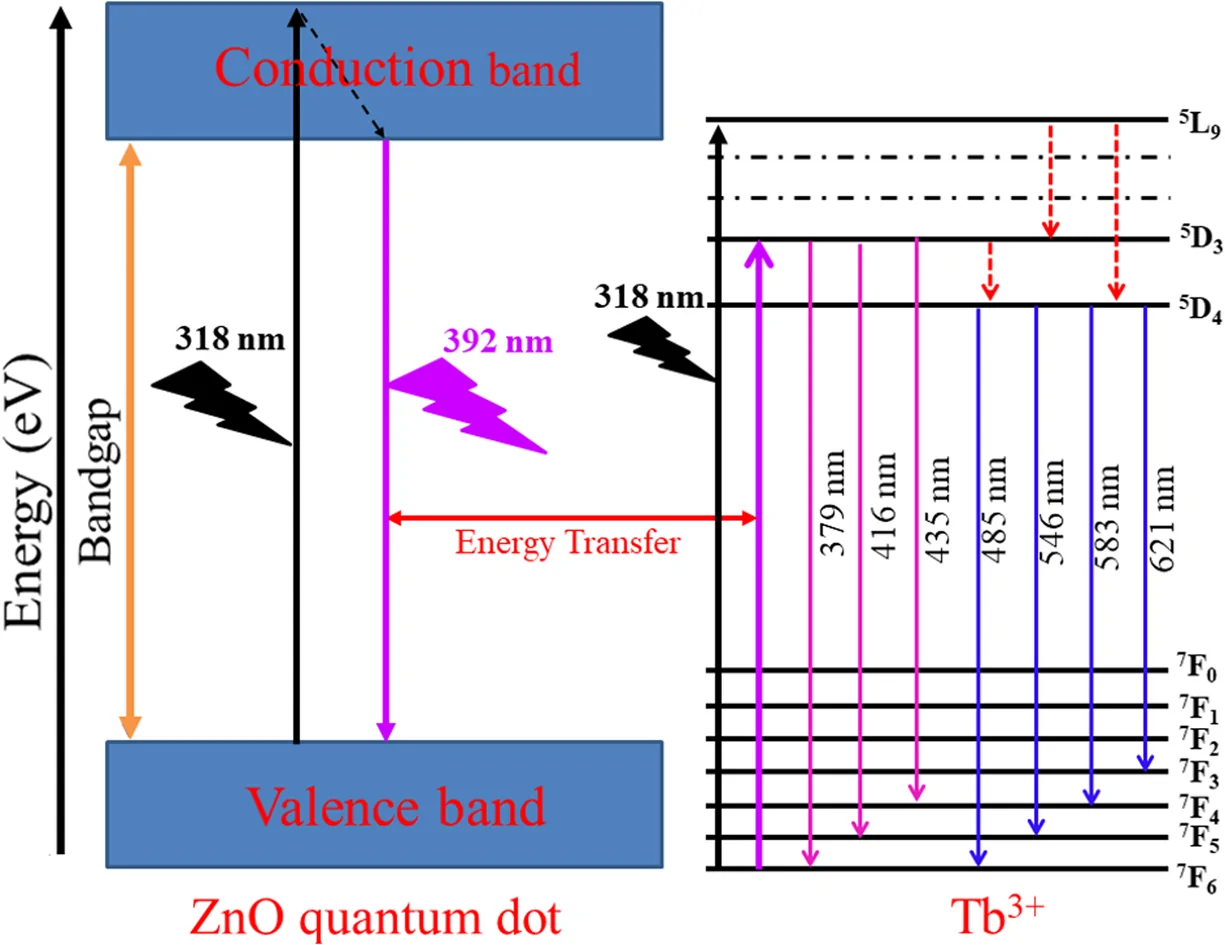

## 1. Introduction

Semiconductor QDs are an important class of nanomaterials due to their size-dependent electronic and optical properties. Tunable band gap widths, broad absorption spectra, narrow emission line widths, and high surface sensitivity enable a wide range of applications in optoelectronics, lasers, photonics, and bioimaging.^1–5^ However, the quantum efficiency of QDs is often limited by factors such as non-radiative recombination due to defects, surface states, and short excited state lifetimes.^6,7^ Therefore, more and more research is being conducted to improve the luminescence efficiency of semiconductor QDs. Furthermore, research is also focused on enhancing optoelectronic and magnetic properties that are absent in pure semiconductor QDs.

In semiconductors, ZnO has significant application potential due to its wide band gap (3.37 eV), large exciton binding energy (60 meV), strong ultraviolet absorption, low toxicity, and high chemical stability.^8,9^ Unlike toxic Cd-based semiconductors, ZnO offers a safer and more environmentally sustainable alternative while exhibiting significant potential for optical and multifunctional applications.^10^ Importantly, ZnO's ability to contain defect states such as oxygen vacancies and intercalated zinc atoms provides further opportunities to fine-tune host-doper energy pathways, as well as magnetic interactions.^11^

Rare earth ion doping has become one of the most effective methods for overcoming the optical limitations of semiconductor QDs while generating highly stable, sharp, and characteristic fluorescence emissions.^12,13^ Tb^3+^ ions are particularly attractive because of their partially filled 4f^8^ configuration, strong green emission from the ^5^D_4_ → ^7^F_5_ transition, long fluorescence lifetime, large magnetic moment, and rich spectral behavior.^14^ These characteristics make Tb^3+^ a dopant capable of altering both the optical and magnetic properties of semiconductor QDs.^15^ Incorporating Tb^3+^ can create efficient luminescence centers, alter emission color, and enhance the lifetime of the radiation for applications in lasers and optical amplifiers.^16^ The large unpaired 4f electronic state of Tb^3+^ can induce ferromagnetic interactions, transforming ZnO, which is inherently diamagnetic, into a dilute magnetic semiconductor.^17^ Simultaneous tuning of optical and magnetic properties is particularly important for the development of multifunctional nanomaterials.

Although Tb^3+^-doped ZnO QDs have many potential applications, no studies have simultaneously addressed their structural characterization, optical properties, Judd–Ofelt spectral parameters, energy transfer mechanisms, and magnetic properties. Most previous studies have focused on individual issues such as optical properties and fabrication technology.^18–20^ Therefore, a comprehensive study of the optical and magnetic properties of Tb^3+^-doped ZnO QDs is essential for both basic and applied research. The main objective of this study is to systematically investigate the effect of Tb^3+^ concentration on the structural, optical, Judd–Ofelt spectroscopic, fluorescence lifetime, and magnetic properties of ZnO QDs to develop multifunctional nanomaterials for photonic, magneto-optical, and spintronic applications.

## 2. Experimental

### 2.1. Materials

Zinc acetate dihydrate (Zn(CH_3_COO)_2_·2H_2_O, 99.999%), terbium nitrate hexahydrate (Tb(NO_3_)_3_·6H_2_O, 99.9%), sodium hydroxide (NaOH, 98%), absolute ethanol, toluene, and deionized water were used as starting materials for the synthesis of ZnO:Tb^3+^ QDs. All chemicals were purchased from Sigma-Aldrich and used without further purification. Deionized water and ethanol were employed as solvents throughout the synthesis process.

### 2.2. Synthesis of Tb^3+^-doped ZnO quantum dots

ZnO:Tb^3+^ QDs with different Tb^3+^ concentrations (0, 0.5, 1, 3, 5, and 7 mol%) were synthesized using a facile wet-chemical precipitation method. Initially, an appropriate amount of zinc acetate dihydrate was dissolved in deionized water under continuous magnetic stirring at room temperature to form a homogeneous precursor solution. Subsequently, calculated amounts of terbium nitrate corresponding to the desired Tb^3+^ doping concentrations were added to the zinc precursor solution and stirred thoroughly to ensure the uniform distribution of Tb^3+^ ions. A sodium hydroxide solution was then added dropwise into the mixed precursor solution under vigorous stirring until the desired pH was achieved, leading to the formation of a white precipitate. The reaction mixture was continuously stirred at room temperature for several hours to promote complete nucleation and growth of ZnO:Tb^3+^ QDs. The obtained precipitates were collected by centrifugation, repeatedly washed with deionized water and ethanol to remove residual impurities, and then dried at 60 °C. Finally, the dried powders were annealed at 400 °C to improve crystallinity and stabilize Tb^3+^ incorporation into the ZnO lattice.

### 2.3. Characterization methods

The crystal structure, phase purity, and lattice parameters of the synthesized ZnO:Tb^3+^ QDs were characterized by X-ray diffraction (XRD) using a Bruker D8 Advance diffractometer equipped with Cu Kα radiation (*λ* = 1.5406 Å) operating at 40 kV. Detailed structural refinement was performed by the Rietveld method using GSAS-II software to evaluate the crystallite size and lattice constants. Elemental composition and spatial distribution of Zn, O, and Tb ions were analyzed using an energy-dispersive X-ray spectrometer (EDX) attached to a FE-SEM system, along with elemental mapping measurements. The chemical composition, oxidation states, and local bonding environments of the constituent elements were investigated by X-ray photoelectron spectroscopy (XPS, Thermo Scientific K-Alpha+) using Al Kα radiation as the excitation source. Optical absorption properties were measured using a Shimadzu UV-3600 UV-vis-NIR spectrophotometer over the wavelength range of 200–800. Photoluminescence (PL) and photoluminescence excitation (PLE) spectra were recorded using a Horiba Fluorolog-3 spectrofluorometer equipped with a xenon lamp. Fluorescence decay curves were measured using a time-correlated single-photon counting (TCSPC) system integrated with a Horiba Fluorolog-3 spectrometer. Room-temperature magnetic properties were investigated using a Lake Shore 7407 vibrating sample magnetometer (VSM) under an applied magnetic field range of ±10 kOe.

## 3. Results and discussion

### 3.1. Structural analyses

**Fig. 1:**
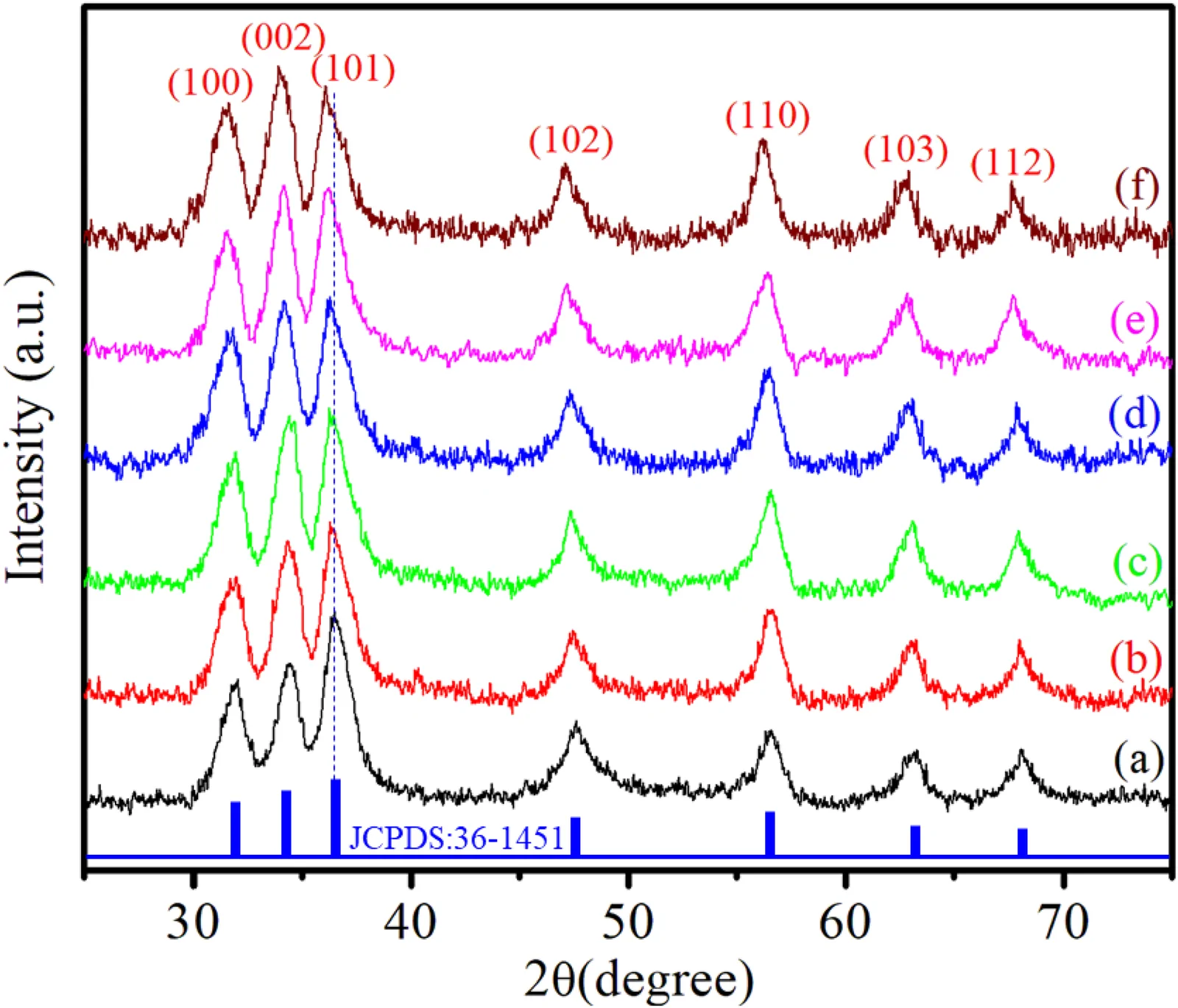
XRD pattern of QDs: (a) ZnO, (b) ZnO:Tb0.5%, (c) ZnO:Tb1%, (d) ZnO:Tb3%, (e) ZnO:Tb5%, and (f) ZnO:Tb7%.

XRD patterns of undoped and Tb^3+^-doped ZnO QDs (0–7%) are shown in Fig. 1. All diffraction peaks can be indexed to the hexagonal wurtzite structure of ZnO (JCPDS no. 36-1451),^21^ with no detectable secondary phases, indicating successful incorporation of Tb^3+^ into the ZnO lattice without forming impurity phases. The diffraction peaks corresponding to the (100), (002), (101), (102), (110), (103), and (112) planes confirm the high crystallinity of the samples. Careful observation of Fig. 1 shows a slight shift of the diffraction peaks towards lower 2*θ* values as the Tb^3+^ concentration increases. This shift shows lattice expansion, consistent with the replacement of Zn^2+^ (0.74 Å) by larger Tb^3+^ ions (∼0.92 Å).^22^

The average crystal size (*D*) of a QD can be calculated based on the Williamson–Hall^23^ or Debye–Scherrer formulas.^6,13^1
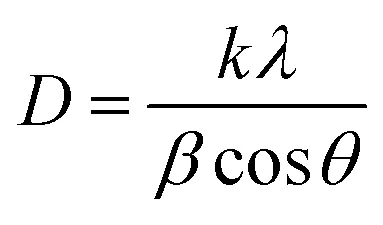


In this equation, *λ* denotes the wavelength of the incident X-ray radiation, *β* is the FWHM of the diffraction peak, and *θ* corresponds to the Bragg diffraction angle. For hexagonal wurtzite crystals, the lattice constants *a* and *c* can be derived from Bragg's law, and their dependence on the Miller indices (*h*, *k*, *l*) is given by:^24^2
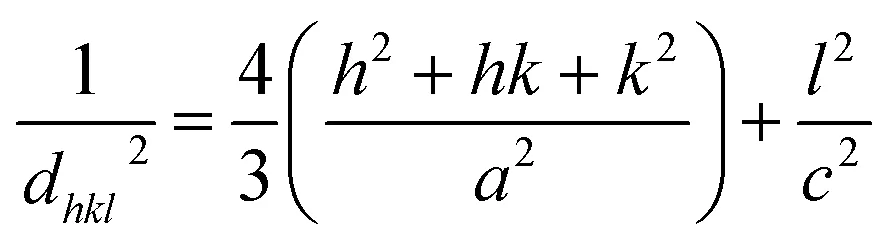
where *d*_*hkl*_ represents the interplanar spacing, which is determined according to Bragg's law:

3 *nλ* = 2*d*_*hkl*_ sin *θ*

The volume of the hexagonal unit cell (*V*) was determined based on the lattice constants *a* and *c* according to the following expression:^24^4
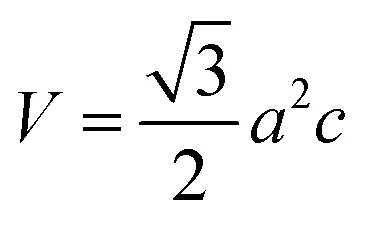


The crystallite strain (*ε*) of the QDs was evaluated from the broadening of diffraction peaks based on the Williamson–Hall approach:^6,13,22^5
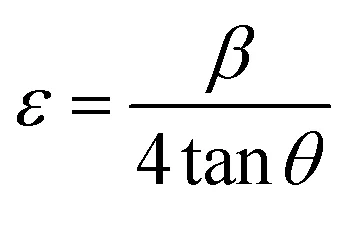


The calculated structural parameters obtained from the XRD analysis, namely the lattice parameters, unit cell volume (*V*), crystallite size (*D*), and microstrain (*ε*), are listed in Table 1.

**Table 1:** Lattice parameters, unit cell volume, crystallite size, and microstrain of Tb^3+^-doped ZnO QDs

Sample	*a* (Å)	*c* (Å)	2*θ*_101_	*β* ×10^−2^ (rad)	Cell volume (Å^3^)	*D* (nm)	*ε* ×10^−3^
ZnO	3.249	5.083	36.47°	3.84	46.465	3.802	29.139
ZnO:Tb0.5%	3.252	5.102	36.41°	3.84	46.727	3.801	29.190
ZnO:Tb1%	3.253	5.126	36.36°	3.87	46.976	3.771	29.461
ZnO:Tb3%	3.255	5.145	36.31°	3.88	47.208	3.761	29.581
ZnO:Tb5%	3.258	5.177	36.23°	3.91	47.589	3.731	29.880
ZnO:Tb7%	3.259	5.245	36.11°	3.91	48.245	3.730	29.986

**Fig. 2:**
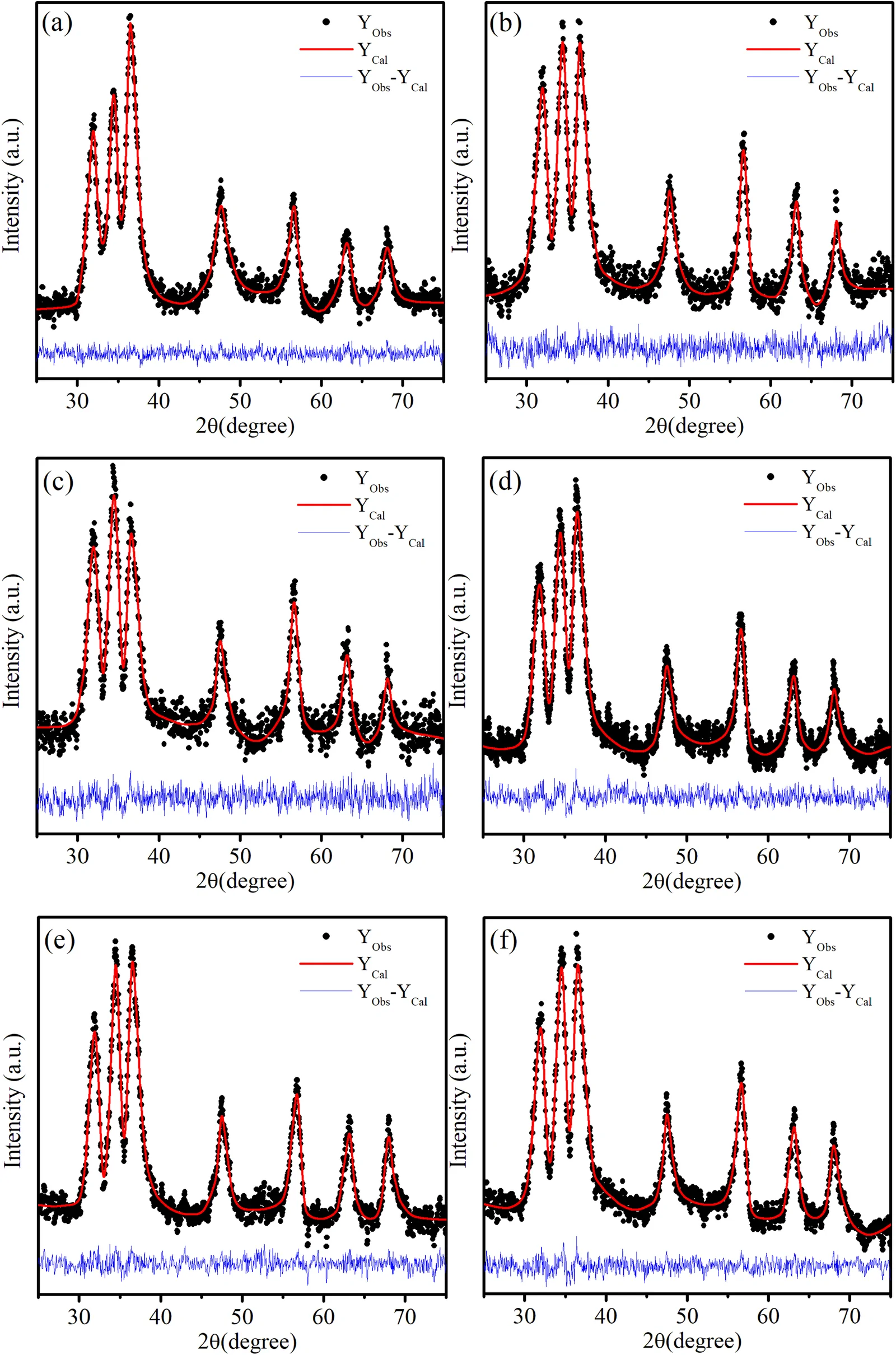
Rietveld refinement of XRD patterns for ZnO and Tb^3+^-doped ZnO QDs at doping concentrations: (a) 0, (b) 0.5, (c) 1, (d) 3, (e) 5, and (f) 7%.

Rietveld refinement plays a crucial role in accurately determining the crystal structure and lattice parameters of Tb^3+^-doped ZnO. This method allows for XRD pattern fitting, providing reliable information on phase purity, lattice distortion, and the integration of Tb^3+^ ions into the ZnO host. Through this analysis, small changes in peak position and intensity can be correlated with structural changes caused by doping. The Rietveld refinement provides conclusive evidence of successful doping. The Rietveld refinement results (Fig. 2) show good agreement between observed and calculated samples, with a low residual error (*Y*_obs_ − *Y*_cal_), confirming the reliability of the structural model. No additional peaks corresponding to Tb-related oxides are observed, suggesting that Tb^3+^ ions are either substitutionally incorporated or located at interstitial sites within the ZnO lattice. The accuracy of the Rietveld refinement was evaluated using the reliability factors *R*_wp_, *R*_p_, *R*_exp_, *R*_F_, and the goodness-of-fit parameter *χ*^2^. The *χ*^2^ value was calculated using *χ*^2^ = (*R*_wp_/*R*_exp_)^2^. The calculated results of the above parameters are observed in Table 2. The obtained low *R* factors and acceptable *χ*^2^ values confirm the good agreement between the observed and calculated XRD patterns. These results indicate that the refinement converged reliably and that the hexagonal wurtzite ZnO structural model is appropriate for the Tb^3+^-doped ZnO QDs.

**Table 2:** Rietveld refinement reliability factors of Tb^3+^-doped ZnO QDs

Sample	*R*_p_ (%)	*R*_wp_ (%)	*R*_exp_ (%)	*R*_F_ (%)	*χ* ^2^
ZnO	12.78	17.14	15.97	4.94	1.15
ZnO:Tb0.5%	14.94	18.45	16.83	6.90	1.20
ZnO:Tb1%	13.39	18.01	16.06	6.12	1.26
ZnO:Tb3%	14.85	18.21	16.75	6.43	1.18
ZnO:Tb5%	13.92	17.15	15.83	5.67	1.17
ZnO:Tb7%	13.03	17.09	15.91	5.82	1.16

The lattice parameter *a* increases from 3.249 to 3.259 Å, while *c* increases from 5.083 to 5.245 Å as the Tb concentration increases, resulting in an expansion of the unit cell volume from 46.465 to 48.245 Å^3^. The increase in lattice parameters is consistent with Vegard's law, confirming the successful doping and the lattice distortion caused by the Tb^3+^ ions.

The crystallite size, calculated using the Scherrer equation, shows a slight decrease from 3.802 to 3.730 nm with increasing Tb content. This reduction suggests that Tb^3+^ ions hinder crystal growth, possibly due to lattice strain and defect formation. Simultaneously, the microstrain (*ε*) increases from 29.139 × 10^−3^ to 29.986 × 10^−3^, indicating enhanced lattice distortion due to the ionic size mismatch and charge imbalance between Tb^3+^ and Zn^2+^. The crystallite size in this work is smaller than that reported in ref. 15 and 17, while the macrostrain/microstrain is higher. This difference is mainly related to the much smaller particle size of our ZnO:Tb^3+^ QDs. The particle size of the present samples is only about 6–8 nm, whereas the reported samples in ref. 15 and 17 are several tens to several hundreds of nanometers in size. The very small particle size causes stronger peak broadening, higher surface disorder, and larger internal strain.

### 3.2. Composition and spatial distribution analyses

To further verify the chemical composition and the incorporation of Tb^3+^ ions into the ZnO lattice, X-ray photoelectron spectroscopy (XPS) analysis was performed. This technique provides detailed information on the elemental composition, oxidation states, and chemical bonding environment of the constituent elements. In particular, XPS is essential for confirming the valence state of Tb ions and distinguishing between substitutional incorporation and surface segregation in ZnO QDs.

**Fig. 3:**
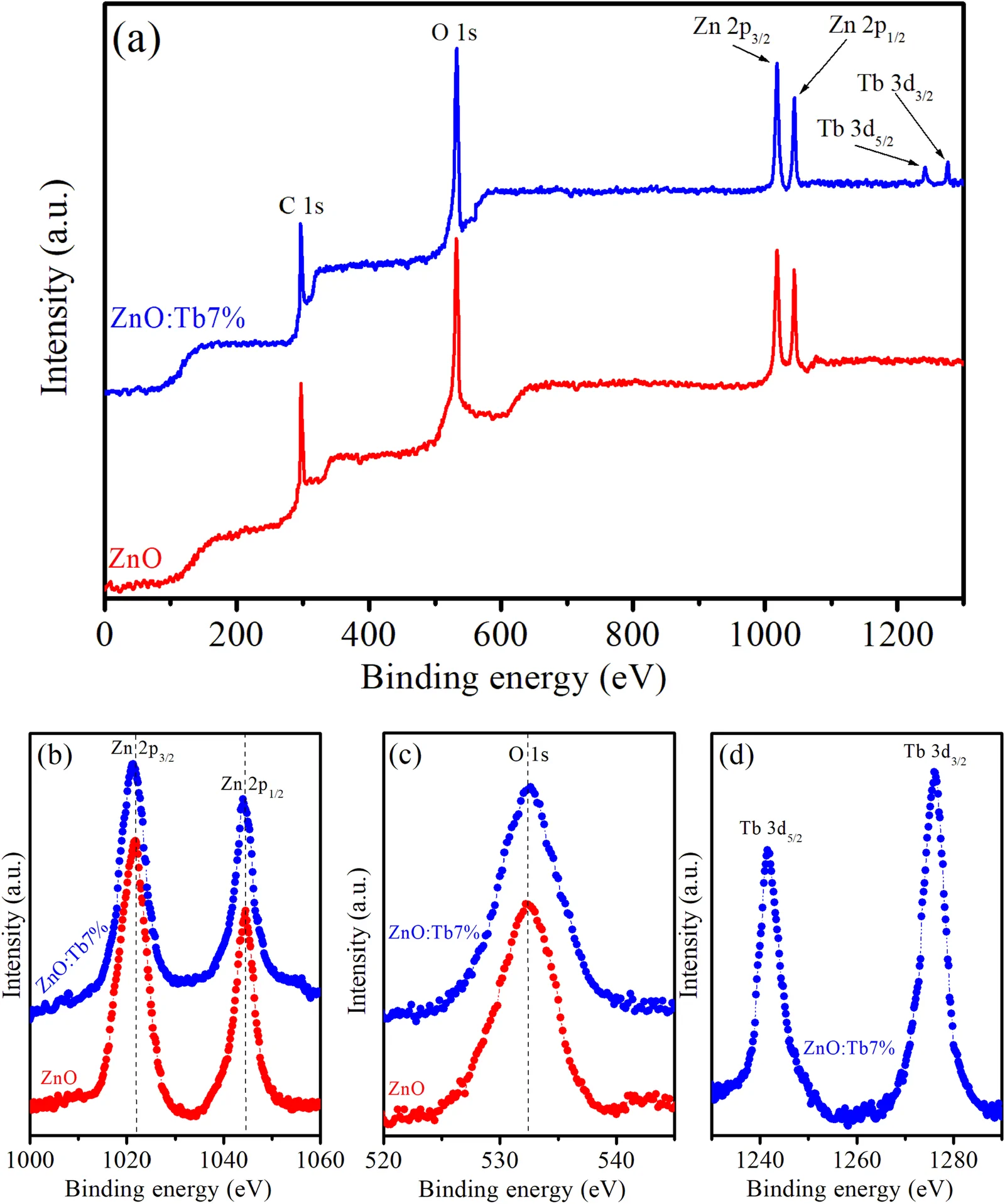
(a) XPS spectrum of ZnO and ZnO:Tb7% QDs. (b–d) High-resolution XPS spectra of Zn, O, and Tb elements.

Fig. 3(a) presents the survey XPS spectra of pure ZnO and ZnO:Tb7% QDs, confirming the presence of Zn, O, and Tb elements in the samples, while the C 1s signal originates from the original precursor. The high-resolution spectra in Fig. 3(b–d) provide further details about the chemical states of the elements in the QDs. For undoped ZnO, the Zn 2p_3/2_ and Zn 2p_1/2_ peaks located at around 1021.9 and 1044.6 eV are characteristic of Zn^2+^ in the ZnO lattice.^25^ In the Tb-doped sample, both Zn 2p peaks shift slightly toward lower binding energy by about 0.22 eV. This negative shift indicates a modification of the local electronic environment around Zn ions after Tb incorporation.^26^ This result can be explained by the replacement of Zn^2+^ with Tb^3+^ causing charge imbalance and lattice distortion, thereby promoting the formation of compensating defects such as oxygen vacancies or local electron donor-like states. These defect states can increase the electron density around neighboring Zn atoms, partially screening the Zn core levels and thereby reducing their binding energy.

In contrast, the O 1s peak of ZnO:Tb7% shifts toward higher binding energy by approximately 0.19 eV relative to pure ZnO.^11,12^ This shift suggests that the oxygen ions experience a different bonding environment in the doped lattice. The incorporation of Tb^3+^, which has a larger ionic radius and a higher valence state than Zn^2+^, can alter the Zn–O bond distribution and increase lattice disorder.^15,17,18^ As a result, part of the oxygen species may become associated with defect-related environments, such as oxygen near distorted Tb–O–Zn linkages, oxygen-deficient regions, or chemically less electron-rich sites.^27^ This leads to a reduction in the local electron density around O atoms, making the O 1s electrons more strongly bound and shifting the O 1s peak to higher binding energy. Therefore, the opposite shifts of Zn 2p and O 1s reflect the redistribution of charge density and local structural distortion caused by Tb doping. The appearance of the Tb 3d_5/2_ and Tb 3d_3/2_ peaks in Fig. 3(d) further confirms the successful incorporation of Tb into the ZnO QDs.^28^ The results observed from the XPS spectrum indicate that Tb^3+^ doping not merely introduces a trivalent ion into the host crystal lattice but also significantly alters the local chemical bonding, defect structure, and electron distribution of ZnO, which is predicted to strongly affect its optical and magnetic properties.

**Fig. 4:**
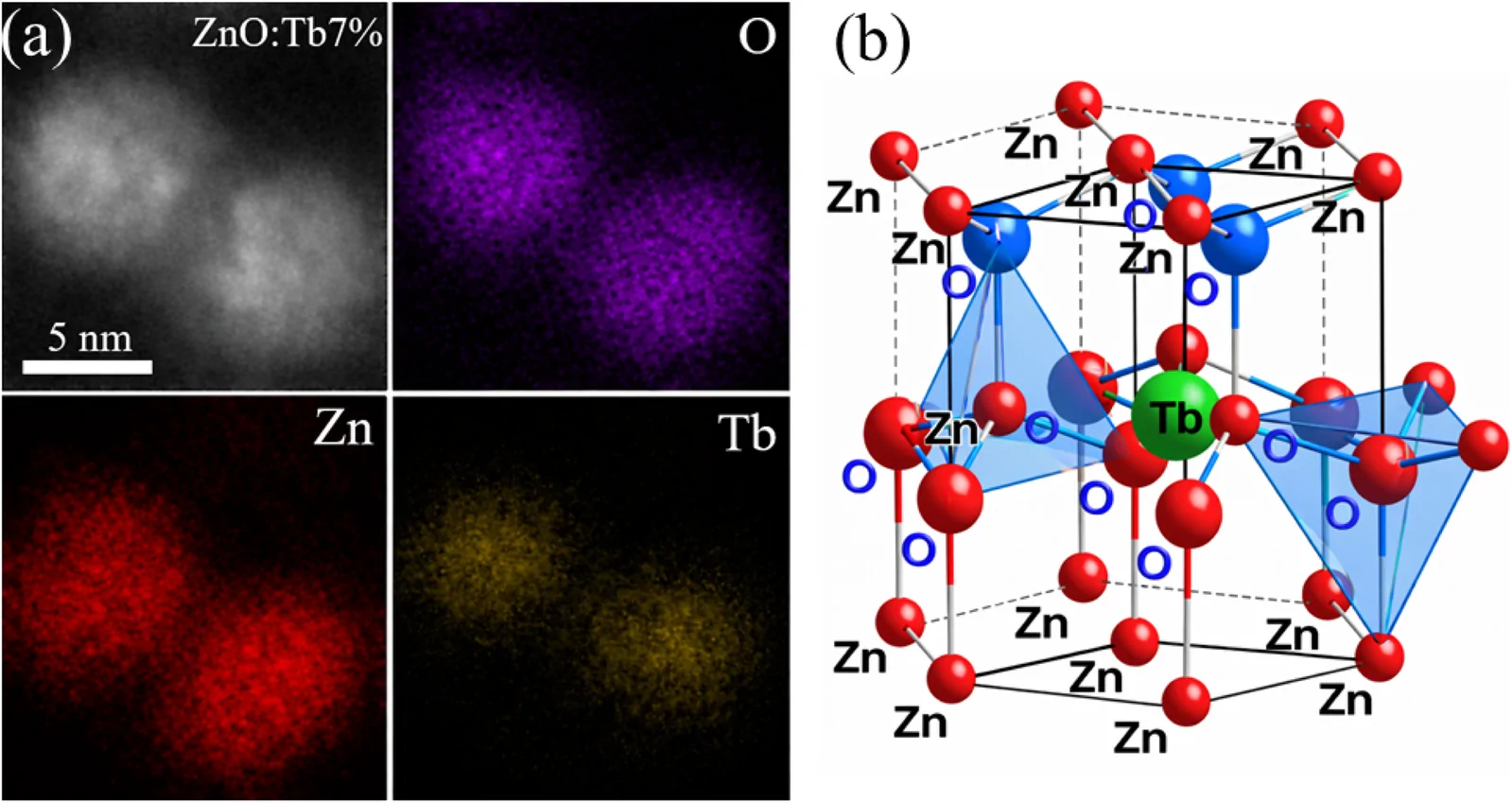
(a) EDX mapping images of ZnO:Tb7% QDs and (b) the unit-cell scheme of ZnO:Tb^3+^ QDs with a wurtzite structure.

To gain deeper insight into the spatial distribution of the constituent elements and verify the homogeneous doping of Tb^3+^ ions, energy-dispersive X-ray (EDX) mapping was carried out. Fig. 4(a) shows the EDX elemental mapping images of ZnO:Tb7% QDs, clearly demonstrating the spatial distribution of Zn, O, and Tb elements. The mapping results reveal that Zn and O are uniformly distributed throughout the nanoparticles, confirming the formation of a homogeneous ZnO matrix. It can be observed that the Tb element is also well dispersed, with no clumping, indicating the successful incorporation of Tb ions into the ZnO QDs. This uniform distribution supports the XPS results and suggests that Tb^3+^ ions are effectively integrated within the ZnO host lattice rather than forming separate clusters. Fig. 4(b) illustrates the schematic unit-cell structure of Tb-doped ZnO with a hexagonal wurtzite configuration. In this structure, Zn atoms are tetrahedrally coordinated with O atoms, forming a stable ZnO lattice. The substitution of Zn^2+^ by Tb^3+^ ions leads to local lattice distortion due to the larger ionic radius and higher valence state of Tb^3+^. This substitution can induce charge imbalance, which is typically compensated by the formation of intrinsic defects such as oxygen vacancies or zinc interstitials.^11,12,15^

### 3.3. Absorption properties

**Fig. 5:**
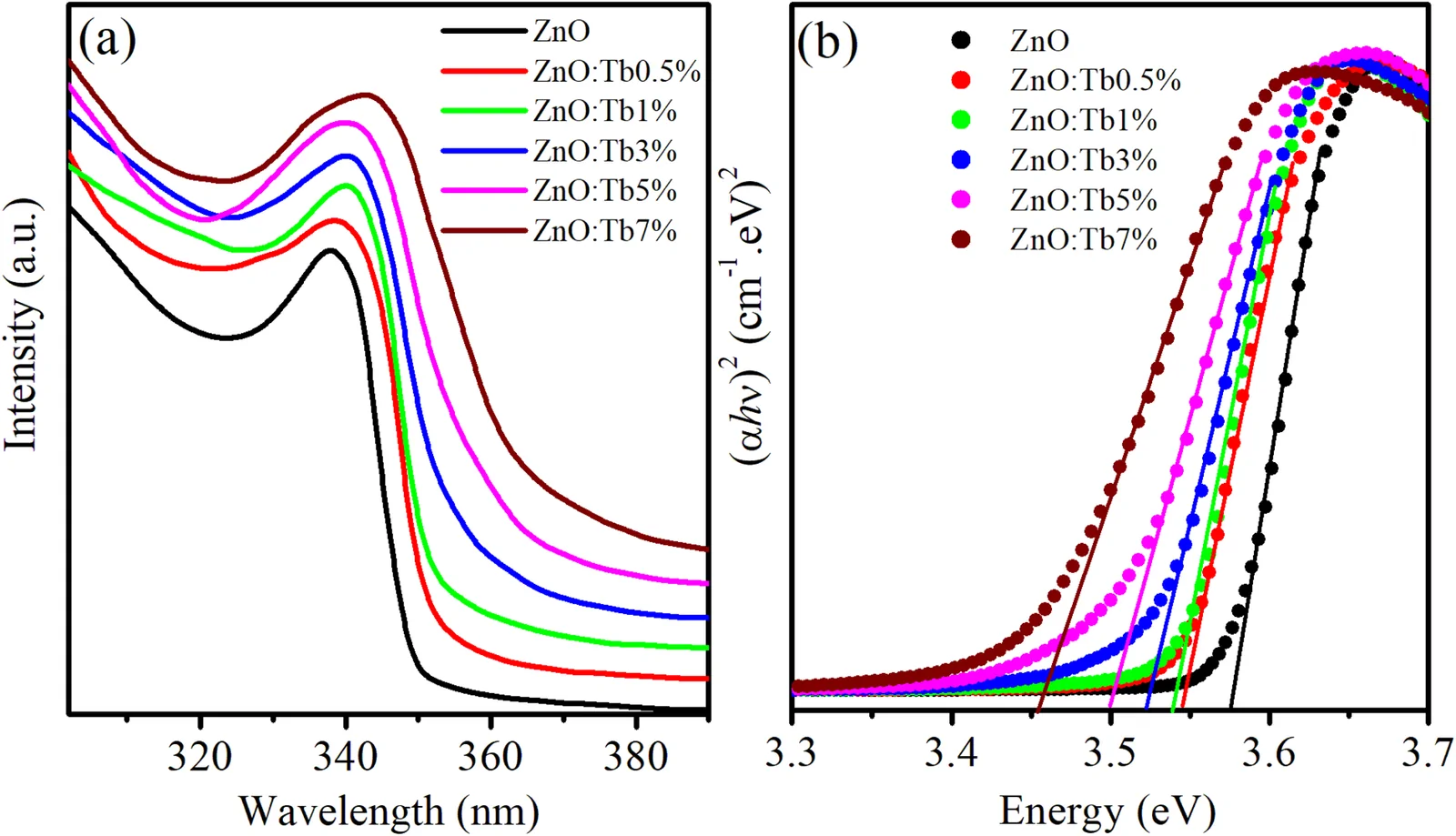
(a) UV-vis absorption spectra of undoped and Tb-doped ZnO QDs and (b) corresponding Tauc plots of (*αhν*)^2^*versus* photon energy (*hν*) for the determination of the direct optical band gap.

Fig. 5(a) presents the UV-vis absorption spectra of pure ZnO and Tb^3+^-doped ZnO QDs with varying doping concentrations (0–7%). Undoped ZnO QDs exhibit a distinct absorption peak at approximately 338 nm, corresponding to the intrinsic band–band transition of ZnO.^12,17^ Compared to bulk ZnO (≈375 nm), the absorption peak is significantly blue-shifted, indicating strong quantum confinement effects associated with the nanoscale size of the QDs.^11,15,18^ Upon Tb^3+^ incorporation, the absorption peak gradually shifts toward longer wavelengths (red-shift), accompanied by a slight broadening of the absorption tail. The positions of the absorption peaks and the bandgap widths of the samples are observed in Table 3. This red-shift can be attributed to the combined effects of lattice distortion induced by Tb^3+^ substitution and the introduction of defect-related electronic states near the band edges.^11,15,18^ Furthermore, the increase in particle size during doping can also contribute to a reduction in quantum confinement, further promoting the red shift. The Tauc plots of (*αhν*)^2^*versus* photon energy (*hν*), shown in Fig. 5(b), were employed to estimate the optical band gap (*E*_g_) of the samples using the Tauc relation:^6,13,22^

6 *αhv* = *A*(*hv* − *E*_g_)^*n*^

**Table 3:** The parameters of samples: Abs peak, *E*_g_, and Urbach energy

Sample	Abs peak (nm)	*E*_g_ (eV)	Diameter (nm)	Urbach energy (meV)
ZnO	338.2	3.57	5.23	35.16
ZnO:Tb0.5%	339.1	3.55	5.71	45.96
ZnO:Tb1%	340.4	3.54	5.76	48.34
ZnO:Tb3%	340.6	3.52	6.06	88.34
ZnO:Tb5%	341	3.50	6.61	96.28
ZnO:Tb7%	343.5	3.45	8.06	102.75

In this relation, *A* is a constant, *α* denotes the absorption coefficient, and *hν* is the photon energy, while *E*_g_ represents the optical band gap. The exponent *n* is determined by the type of electronic transition. Since ZnO is a direct band gap semiconductor, the exponent *n* = 1/2 was used. The extrapolation of the linear region of the curves to the energy axis yields the band gap values. Undoped ZnO QDs exhibit an *E*_g_ of approximately ∼3.57 eV, which is higher than that of bulk ZnO (3.37 eV) due to quantum confinement effects. With increasing Tb^3+^ concentration, the band gap decreases progressively, indicating a systematic narrowing of *E*_g_. This behavior can be explained by the incorporation of Tb^3+^ ions into the ZnO lattice, which induces lattice strain and generates defect states within the forbidden band. These defect states act as intermediate energy levels, effectively reducing the optical band gap.^11,15,17^ Moreover, the increased structural disorder and electron–phonon interactions in doped samples also contribute to the broadening of the absorption peaks. The results demonstrate that Tb^3+^ doping effectively modulates the optical absorption of ZnO QDs, enabling tunable optical properties.^12,17^

The average size of ZnO QDs can be estimated based on the energy position of the first excitonic transition, corresponding to the lowest-energy ^1^S(h)–^1^S(e) excitonic transition. This estimation is commonly carried out using the effective mass approximation (EMA) model, as described in eqn (7).^29^7
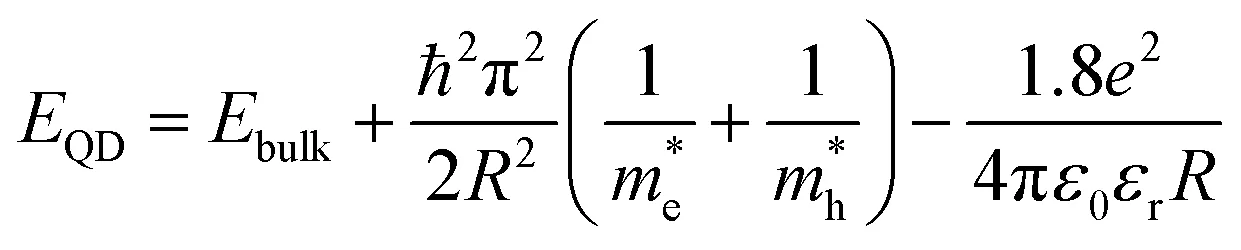


In this model, the bandgap energy of the QDs, denoted as *E*_QD_, depends on the particle radius *R*, reflecting the quantum confinement effect. The parameter *E*_bulk_ represents the bandgap energy of bulk cubic ZnO (approximately 3.37 eV), which serves as a reference value in the absence of confinement. Here, *ℏ* is the reduced Planck constant. The effective masses of the charge carriers are taken as 
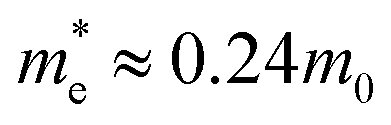
 for electrons and 
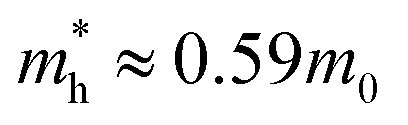
 for holes, where *m*_0_ is the free electron mass. *ε*_0_ and *ε*_r_ represent the vacuum permittivity and the relative dielectric constant of ZnO, respectively, with *ε*_*r*_ ≈ 8.5.^30^ The elementary charge is denoted by *e*. The sizes of the QDs were determined and are given in Table 3.

The data presented in Table 3 clearly reveal a systematic increase in the average particle size of ZnO QDs with increasing Tb doping concentration, from 5.23 nm for undoped ZnO to 8.06 nm for the ZnO:Tb7% sample. This trend, along with the redshift of the absorption edge and a gradual decrease in the optical bandgap from 3.572 eV to 3.454 eV, indicates a weakening of the quantum confinement effect as the particle size increases. The observed size enlargement of ZnO QDs upon Tb incorporation can be attributed to two main factors. First, the introduction of Tb^3+^ ions, which possess a larger ionic radius compared to Zn^2+^, induces lattice distortion and modifies the nucleation and growth kinetics during the synthesis process. This effect tends to suppress the formation of numerous small nuclei while promoting the growth of larger crystallites. Second, at higher Tb concentrations, enhanced interparticle interactions and aggregation tendencies may occur, leading to an increase in the particle size.

The Urbach energy (*E*_u_) provides important insight into the degree of structural disorder and the density of localized states near the band edge in semiconductor QDs.^31^ In ZnO:Tb QDs, *E*_u_ is commonly extracted from the linear region of the ln(*α*) *versus hν* plots and reflects the extent of band-tail states induced by lattice imperfections, defect centers, and compositional disorder, as determined from the equation:^32^8
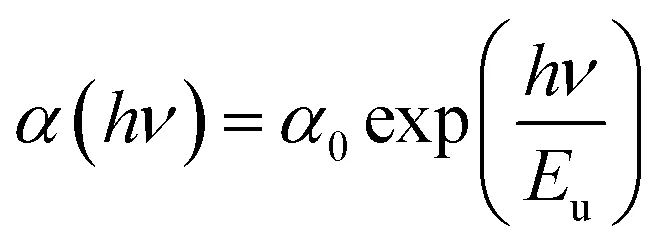

9 ln(*α*) = ln(*α*_0_) + *hν*/*E*_u_

**Fig. 6:**
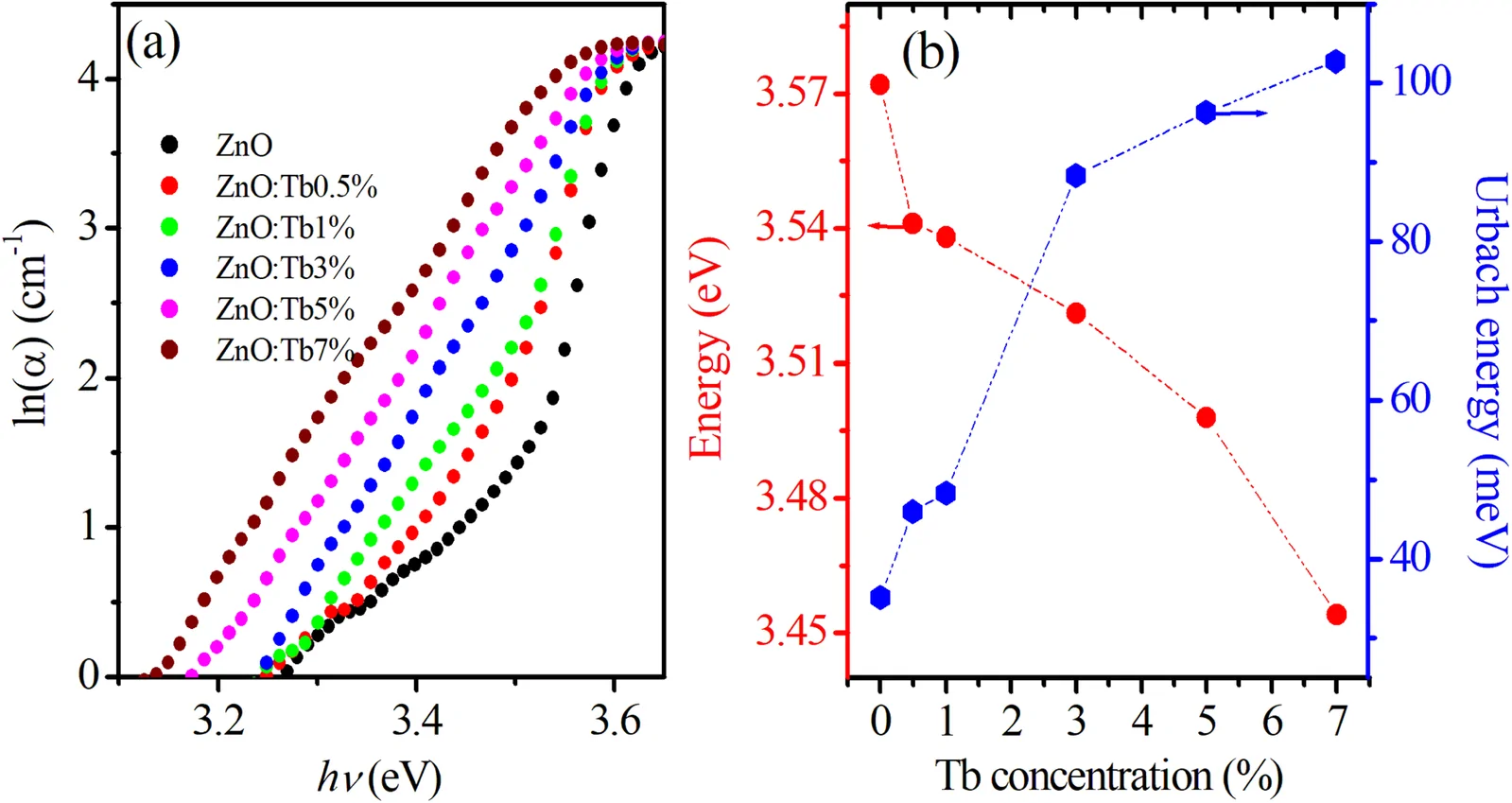
(a) Urbach plots for samples. (b) The plot of *E*_g_ and Urbach energy with Tb concentrations.

As shown in Fig. 6(a), the absorption edge becomes progressively broader with increasing Tb content, indicating a pronounced enhancement of the absorption tail. Fig. 6(b) and Table 3 reveal that the Urbach energy increases from 35.16 meV for undoped ZnO to 102.75 meV for ZnO:Tb7%, while the optical bandgap decreases from 3.572 eV to 3.454 eV. This trend clearly suggests that Tb incorporation gradually increases the disorder in the ZnO QDs and promotes the formation of localized electronic states extending into the bandgap.^12,18^ The increase in *E*_u_ with Tb concentration can be mainly attributed to two factors. First, the substitution or incorporation of Tb^3+^ ions into the ZnO host creates lattice distortion because of the mismatch in ionic size and valence state between Tb^3+^ and Zn^2+^, thereby increasing strain and perturbing the local crystal field. Second, higher Tb concentrations tend to generate a larger number of intrinsic and extrinsic defect states, such as oxygen-related defects, Zn vacancies, and dopant-associated defect complexes, which broaden the band tail and enhance sub-bandgap absorption. Therefore, the continuous rise in Urbach energy confirms that Tb doping not only modifies the electronic structure of ZnO QDs but also induces increasing structural and energetic disorder, which is consistent with the observed bandgap narrowing and weakening of quantum confinement at higher dopant concentrations.

### 3.4. Luminescence properties and the energy transfer

**Fig. 7:**
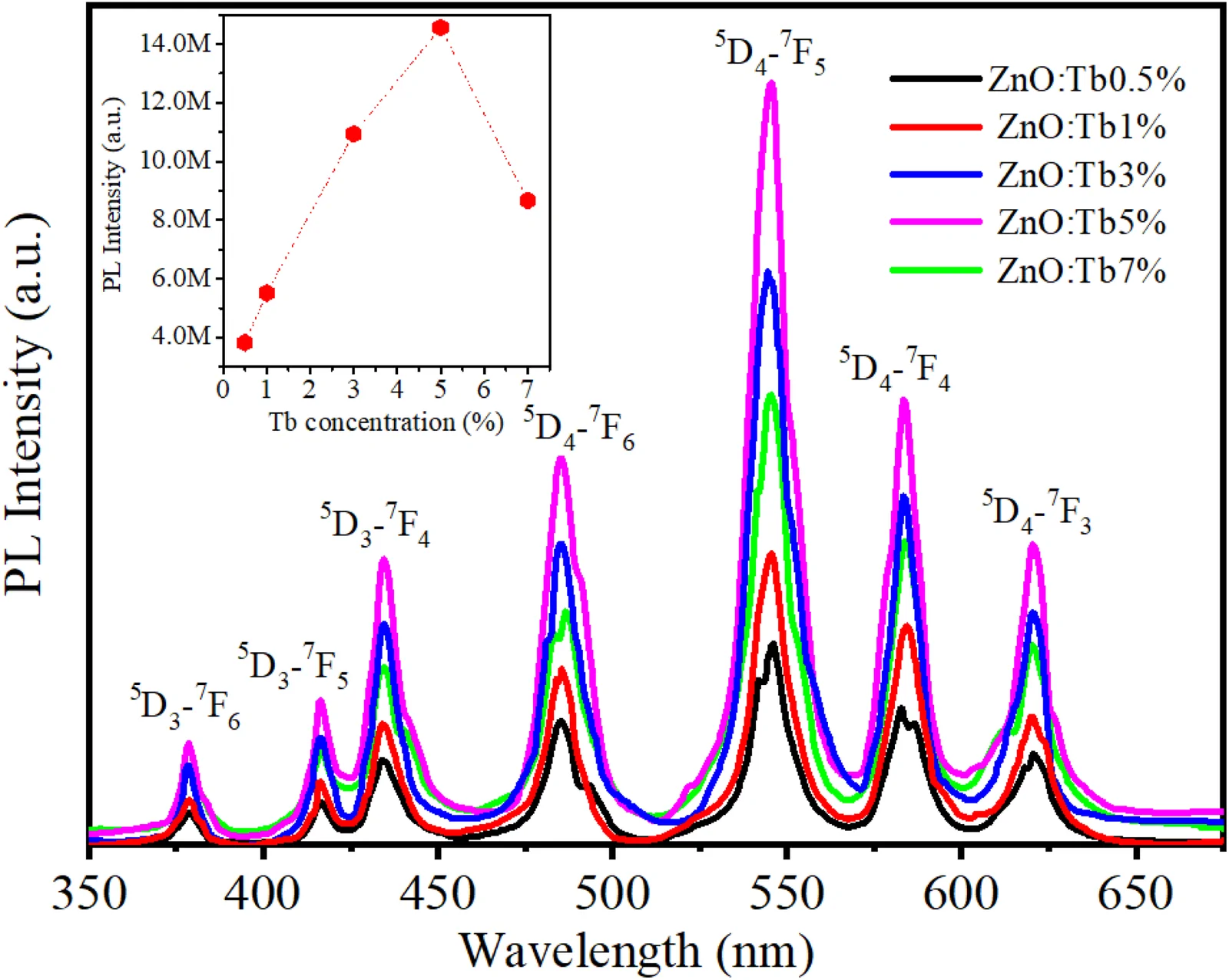
PL spectra of Tb-doped ZnO QDs were excited at 318 nm (^7^F_6_–^5^H_7_ transition).

Fig. 7 shows the luminescence spectra of Tb^3+^-doped ZnO QDs excited at a wavelength of 318 nm. The PL spectra only show the characteristic 4f internal emission of Tb^3+^, indicating that rare earth ions act as the main luminescence centers in this system. The emission bands observed at approximately 379, 416, and 435 nm are assigned to the ^5^D_3_ → ^7^F_6_, ^5^D_3_ → ^7^F_5_, and ^5^D_3_ → ^7^F_4_ transitions, respectively, whereas the stronger bands centered at about 485, 546, 583, and 621 nm originate from the ^5^D_4_ → ^7^F_6_, ^5^D_4_ → ^7^F_5_, ^5^D_4_ → ^7^F_4_, and ^5^D_4_ → ^7^F_3_ transitions, respectively.^19,22^ Among them, the green emission at ∼546 nm corresponding to the ^5^D_4_ → ^7^F_5_ transition is the most intense feature, which is a typical fingerprint of Tb^3+^ and confirms that radiative relaxation from the ^5^D_4_ level is the dominant recombination pathway.^22,27^ A noteworthy point is that the emissions from the ^5^D_3_ level remain observable but are much weaker than those from ^5^D_4_, suggesting efficient non-radiative relaxation from ^5^D_3_ to the lower-lying ^5^D_4_ level, as commonly found in Tb^3+^-activated systems. This intense and spectrally sharp green emission makes ZnO:Tb^3+^ QDs promising for green phosphors, display devices, and solid-state lighting.

The inset reveals that the PL intensity increases with Tb^3+^ concentration up to 5% and then decreases at 7%, indicating typical concentration-dependent activation and quenching behavior. The primary reason for the increased emission intensity is the gradual increase in the number of optically active Tb^3+^ luminescent centers, which improves the ability to capture excitation and recombine subsequent radiation through Tb^3+^-related levels. On the other hand, higher Tb^3+^ concentrations can facilitate energy transfer from the host to the activator, thus increasing the number of ^5^D_4_ emission states.^19,26^ The reason for the decrease in emission intensity at concentrations greater than 5% is quenching, as concentration becomes significant when the mean distance between Tb^3+^ ions decreases. Under such conditions, non-radiative energy migration among adjacent Tb^3+^ ions is promoted, allowing the excitation energy to reach quenching centers such as structural defects, surface states, dangling bonds, or hydroxyl-related species, where it is dissipated non-radiatively.^19,22,26^ In addition, strong Tb^3+^–Tb^3+^ interactions may induce cross-relaxation and local lattice distortion, both of which further suppress the emission intensity at excessive dopant concentrations.

To further understand the concentration quenching mechanism, the critical distance (*R*_c_) between Tb^3+^ ions was estimated using Blasse's equation:^33^10
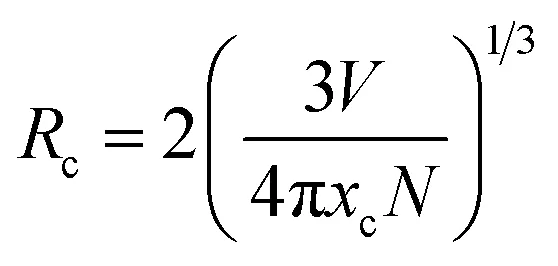
where *V* is the unit-cell volume, *x*_c_ is the critical activator concentration, and *N* is the number of available cation sites in the unit cell. In this work, the PL intensity reaches its maximum at 5 mol% Tb^3+^, so *x*_c_ = 0.05. For the ZnO:Tb5% sample, *V* = 47.589 Å^3^, and *N* = 2 for the wurtzite ZnO unit cell, so *R*_c_ = 9.69 Å. The calculated *R*_c_ value is larger than 5 Å, indicating that exchange interaction is not the dominant mechanism for concentration quenching. According to Dexter's theory, when *R*_c_ > 5 Å, concentration quenching is mainly governed by electric multipolar interaction among activator ions. Therefore, the quenching observed at 7 mol% Tb^3+^ can be attributed to enhanced multipolar interaction and non-radiative energy migration between neighboring Tb^3+^ ions. The excitation energy can migrate through Tb^3+^ ions and finally reach defect-related quenching centers, leading to the decrease in PL intensity.

**Fig. 8:**
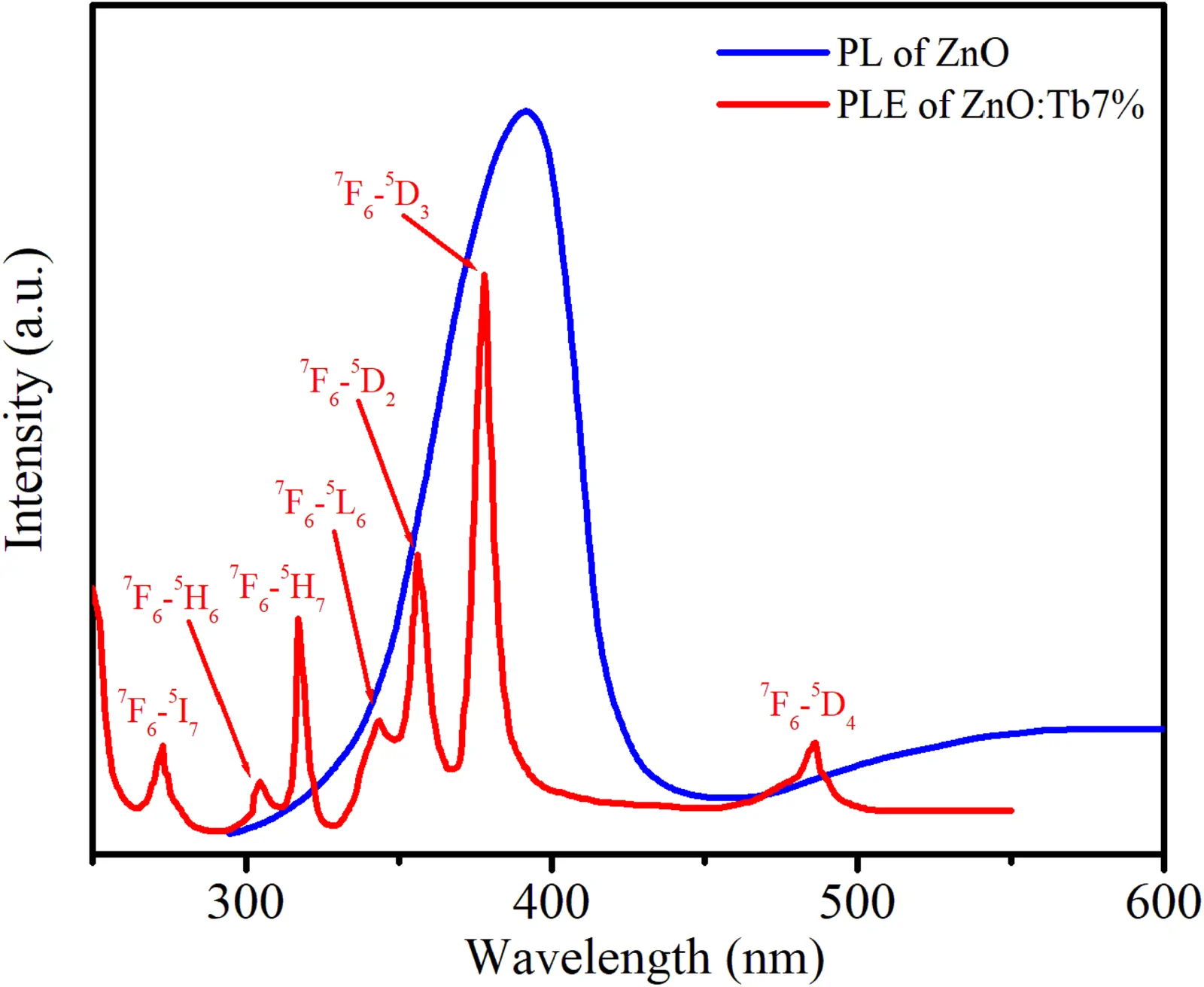
PL spectrum of ZnO and PLE spectrum of ZnO:Tb7% QDs.

**Fig. 9:**
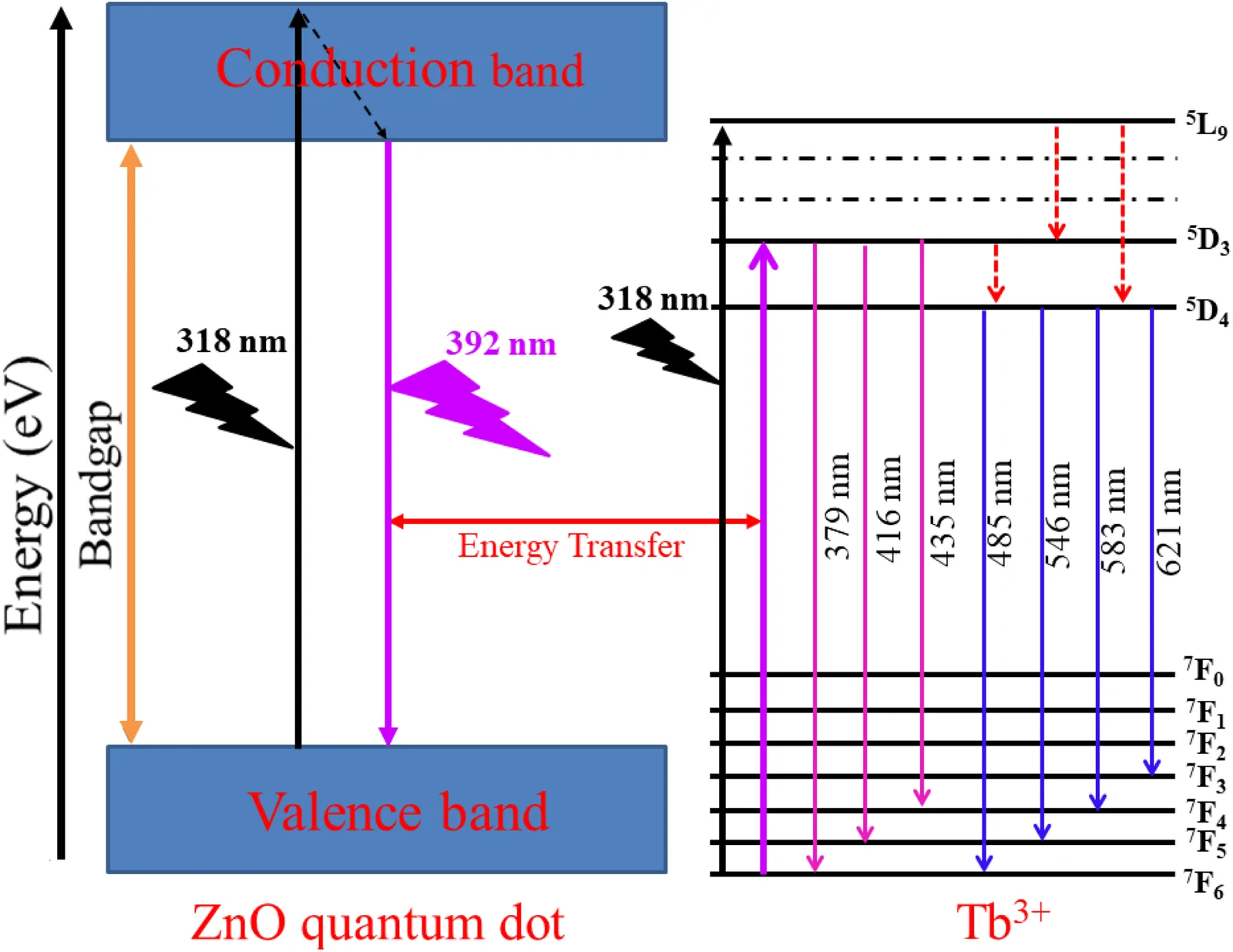
Schematic energy level diagram and the ET process from the ZnO host to Tb ions.

Interestingly, no emission of the ZnO host (also known as exciton recombination) was observed at wavelengths around 400 nm. This result can be explained by several factors: (i) an energy transfer process from the ZnO host to the Tb^3+^ ions is highly effective: after excitation of the ZnO host, the absorbed energy can be transferred to the excited states of Tb^3+^, which then radiate through their characteristic 4f–4f transitions, thus suppressing or masking the host's emission; (ii) the incorporation of Tb^3+^ can alter the defect structure, creating trap channels that favor the localization of excitation on Tb^3+^ over radiative recombination within the host; (iii) in QDs, the large surface area-to-volume ratio increases the non-radiative losses associated with the surface, which can significantly attenuate the natural ZnO emission. Among the above causes, the efficient energy transfer process from the ZnO host to the Tb^3+^ ions is the main cause and is explained in detail in Fig. 8 and 9.

Fig. 8 shows the PL spectrum of ZnO QDs and the photoluminescence excitation (PLE) spectrum of ZnO:Tb7% QDs. To obtain the PLE spectrum, the emission wavelength was fixed at 546 nm, corresponding to the ^5^D_4_ → ^7^F_5_ transition of Tb^3+^ ions. Fig. 8 provides direct evidence for the optical coupling between the ZnO host and Tb^3+^ activators through the comparison of the PL spectrum of undoped ZnO and the PLE spectrum of ZnO:Tb7% QDs. The PL spectrum of ZnO exhibits a dominant emission centered at approximately 392 nm, which can be attributed to excitonic recombination.^15,17^ In the PLE spectrum of ZnO:Tb7%, several sharp excitation bands are clearly observed at around 273, 304, 318, 343, 356, 378, and 486 nm, which are assigned to the intra-4f transitions ^7^F_6_ → ^5^I_7_, ^7^F_6_ → ^5^H_6_, ^7^F_6_ → ^5^H_7_, ^7^F_6_ → ^5^L_6_, ^7^F_6_ → ^5^D_2_, ^7^F_6_ → ^5^D_3_, and ^7^F_6_ → ^5^D_4_, respectively.^17–19^ Among these excitation lines, the transitions near 318, 356, and especially 378 nm are the most intense, revealing that these channels are the most efficient routes for populating the emitting states of Tb^3+^. A particularly important feature is that three excitation bands of Tb^3+^, centered at about 343, 356, and 378 nm, lie within the broad emission envelope of ZnO centered at 392 nm. This spectral overlap is highly significant because it strongly supports the possibility energy transfer from ZnO to Tb^3+^ ions. In other words, the emission energy generated by the ZnO host can be resonantly reabsorbed by Tb^3+^ ions through these overlapping 4f excitation channels.

Under excitation at 318 nm, the photoluminescence behavior of the ZnO:Tb^3+^ QDs is governed by two complementary excitation pathways, namely an indirect ZnO host-sensitized process and a direct f–f excitation of Tb^3+^ ions, as illustrated in Fig. 9. In the host-mediated pathway, the incident photons are primarily absorbed by the ZnO host, promoting electrons from the valence band (VB) to the conduction band (CB) and generating electron–hole pairs. The excited electrons subsequently relax nonradiatively to the bottom of the CB, followed by radiative recombination with holes in the VB, giving rise to an emission centered at approximately 392 nm. Notably, this emission is consistent with the internal 4f absorption transitions of Tb^3+^ ions, particularly the ^7^F_6_ → ^5^D_3_ transition, thus enabling efficient energy transfer (ET) from the ZnO host to the Tb^3+^ activators. As a result, the initial excitation energy is absorbed by the ZnO host and efficiently transferred to the Tb^3+^ ions. From the ^5^D_3_ energy level, Tb^3+^ ions undergo rapid non-radiative relaxation *via* multiphonon processes to the lower ^5^D_4_ energy level, due to the relatively small energy gap between these states and the high phonon coupling in the host crystal lattice. As a result, emissions originating from ^5^D_3_ (*e.g.*, transitions to ^7^F_*J*_ levels in the UV-blue region around 379–435 nm) are significantly suppressed. In contrast, the ^5^D_4_ level acts as the dominant radiative center, giving rise to the characteristic green emissions corresponding to the ^5^D_4_ → ^7^F_*J*_ transitions, with prominent peaks observed at approximately 485 nm (^7^F_6_), 546 nm (^7^F_5_), 583 nm (^7^F_4_), and 621 nm (^7^F_3_), among which the 546 nm emission is typically the most intense due to its favorable electric dipole transition probability.^15,19^

Simultaneously, a direct excitation channel of Tb^3+^ ions is established under 318 nm radiation. Specifically, Tb^3+^ ions can directly absorb excitation photons *via* 4f–4f transitions, moving electrons from the ground state ^7^F_6_ to higher excited states ^5^L_9_. Following this excitation, the electrons rapidly relax non-radiatively to the ^5^D_3_ and ^5^D_4_ levels through a series of multiphonon relaxation processes. From the ^5^D_3_ and ^5^D_4_ levels, the electrons return to the ^7^F_*J*_ levels and emit visible radiation as observed in Fig. 7. Therefore, the coexistence of these two excitation mechanisms (host-sensitized energy transfer from ZnO and direct excitation of Tb^3+^ ions) not only improves the excitation efficiency in the UV region but also ensures effective population of the ^5^D_4_ emitting level. This dual-channel excitation mechanism is crucial for achieving strong and characteristic Tb^3+^ emissions in ZnO QDs and highlights the role of ZnO as an efficient sensitizer for rare-earth luminescence. CIE chromaticity coordinates and color purity are commonly used to evaluate the emission color quality of rare-earth-doped phosphors and luminescent materials for lighting and display applications.^34–36^ Therefore, the strong Tb^3+^ green emission observed in ZnO:Tb^3+^ QDs suggests their potential for color-tunable photonic devices.

### 3.5. Judd–Ofelt theory

In the Tb^3+^ ion, the optical transitions are primarily 4f^8^ intraconfigurational transitions. These transitions are forbidden by Laporte's rule but still occur because the crystal field mixes the 4f state with configurations of varying even/odd properties. Judd–Ofelt theory allows the magnitude of these transitions to be described through three parameters *Ω*_2_, *Ω*_4_, and *Ω*_6_. Three parameters reflect the degree of asymmetry, covalency, and the local environment around the Tb^3+^ ion. The intensity of the transition in rare earth ions can be estimated by the vibration intensity obtained from the absorption spectrum using the following formula:^37,38^11
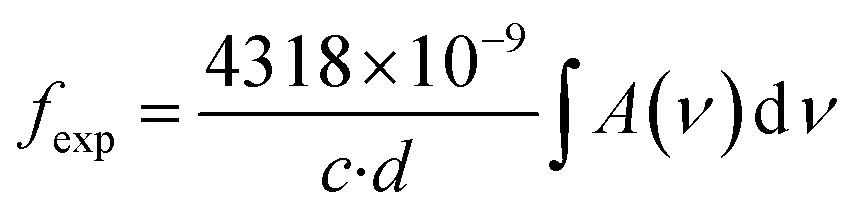
where *A* is the absorbance at energy *ν*, *c* is the concentration; and *d* is the thickness of the sample. In this study, the excitation spectra (PLE) were employed instead of direct absorption measurements due to the intrinsically weak 4f–4f absorption transitions of Tb^3+^ ions, which are often obscured by the strong band-to-band absorption of ZnO. As observed in Fig. 5, the characteristic absorption peaks of Tb^3+^ are absent in the absorption spectra of the samples. To ensure the reliability of the Judd–Ofelt analysis, the PLE spectra were corrected for the instrumental response of the excitation source and detector, and the integrated intensities of the Tb^3+^ excitation bands were normalized. The integrated intensities of the Tb^3+^ excitation bands were normalized with respect to the ^7^F_6_–^5^D_4_ transition, which was chosen as the reference due to its relatively strong and well-resolved feature in the PLE spectrum. This normalization allows the relative oscillator strengths of different transitions to be reliably compared for the Judd–Ofelt analysis.

According to J–O theory, the theoretical oscillator intensity of the electric-dipole transition is determined by the equation:^37,38^12
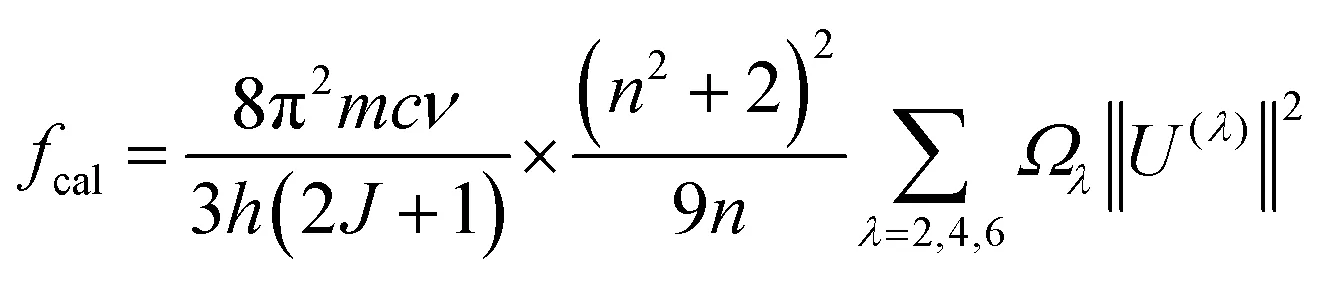
where *n* is the refractive index of the host material, *ν* is the mean wavenumber of the transition, *J* is the total angular momentum of the initial level, *Ω*_*λ*_ are Judd–Ofelt parameters, and ‖*U*^*λ*^‖^2^ are the squared matrix elements of the unit tensor operator of the rank *λ* = 2, 4, and 6.

The J–O parameters are determined by the least squares method using the experimental and theoretical oscillator intensities (solve the *f*_cal_ = *f*_exp_ equation system). The intensity parameters for the ZnO:Tb^3+^ QDs were determined and are summarized in Table 4, along with a comparison with those reported for other hosts. The Judd–Ofelt intensity parameters *Ω*_2_, *Ω*_4_, and *Ω*_6_ provide important information on the local structure and optical behavior of Tb^3+^ ions in different host matrices. Among them, *Ω*_2_ is strongly related to the local asymmetry and covalent character of the Tb–O bonding environment, while *Ω*_4_ and *Ω*_6_ are generally associated with the rigidity, viscosity, and long-range structural properties of the host lattice.

**Table 4:** The intensity parameters of Tb^3+^ ions for some matrices

Host	*Ω*_2_ (10^−20^ cm^2^)	*Ω*_4_ (10^−20^ cm^2^)	*Ω*_6_ (10^−20^ cm^2^)	*Ω*_4_/*Ω*_6_	Ref.
ZnO:Tb0.5%	3.65	2.48	3.12	0.79	This work
ZnO:Tb1%	3.83	2.65	3.53	0.75	This work
ZnO:Tb3%	4.09	2.93	3.88	0.76	This work
ZnO:Tb5%	4.57	2.57	3.42	0.75	This work
ZnO:Tb7%	4.21	2.75	3.64	0.76	This work
Tb:KYb(WO_4_)_2_	1.91	2.41	4.91	0.49	39
K_2_YF_5_:Tb^3+^	2.33	2.29	1.41	1.62	40
CaF_2_:Tb^3+^	4.57	0.49	3.49	0.14	41
TbP_5_O_14_	3.77	1.78	3.43	0.52	42
ZnSe:1%Tb^3+^	4.81	3.47	2.64	1.31	43
NaPO_3_·BaF_2_·Tb^3+^	2.76	3.21	3.36	0.96	44
KYF_4_:Tb^3+^	3.69	1.13	8.68	0.13	45

For the ZnO:Tb^3+^ samples, *Ω*_2_ increases from 3.65 for ZnO:Tb0.5% to 4.57 for ZnO:Tb5% and then slightly decreases to 4.21 for ZnO:Tb7%. This trend indicates that increasing the Tb^3+^ concentration initially enhances the local distortion around Tb^3+^ ions. This can be attributed to the substitution of Zn^2+^ by Tb^3+^, which causes charge imbalance, lattice distortion, and defect formation.^18,19^ The maximum *Ω*_2_ value at 5% suggests that this composition provides the most asymmetric and covalent local environment for Tb^3+^ ions. However, the decrease at 7% may be related to Tb–Tb interaction, local clustering, or concentration-induced structural relaxation.^15,18^ The *Ω*_4_ values vary from 2.48 to 2.93, showing a weaker concentration dependence than *Ω*_2_. The highest *Ω*_4_ value is observed for ZnO:Tb3%, suggesting a relatively enhanced lattice rigidity or local structural stability at this concentration. Meanwhile, *Ω*_6_ increases from 3.12 to 3.88 as the Tb^3+^ concentration increases from 0.5% to 3%, and then it decreases at 5% and partially recovers at 7%. This behavior implies that the crystal-field environment is modified by Tb incorporation but remains relatively stable within the investigated concentration range. The ratio *Ω*_4_/*Ω*_6_ for ZnO:Tb^3+^ remains almost constant, ranging from 0.75 to 0.79. This nearly constant ratio suggests that the relative contribution of the structural-altering effects around the Tb^3+^ ions is preserved despite increasing doping concentrations. Therefore, although Tb^3+^ doping significantly alters the local asymmetry, the overall structural framework of ZnO remains structurally stable.

Compared with other matrices, the *Ω*_2_ values of ZnO:Tb^3+^ are higher than those of Tb:KYb(WO_4_)_2_ and K_2_YF_5_:Tb^3+^, comparable to those of TbP_5_O_14_, CaF_2_:Tb^3+^, and KYF_4_:Tb^3+^, and lower than that of ZnSe:1%Tb^3+^.^39–45^ This suggests that Tb^3+^ ions in ZnO experience a more asymmetric and more covalent environment than in many fluoride and tungstate hosts. Fluoride and tungstate hosts usually exhibit lower *Ω*_2_ values because of their more ionic bonding character and lower local polarizability. The relatively large *Ω*_4_ values of ZnO:Tb^3+^, especially compared with CaF_2_:Tb^3+^ and KYF_4_:Tb^3+^, indicate that the ZnO lattice provides a stronger intermediate-range crystal-field contribution. Meanwhile, the *Ω*_6_ values of ZnO:Tb^3+^ are moderate, being lower than those of KYF_4_:Tb^3+^ but comparable to those of TbP_5_O_14_ and NaPO_3_·BaF_2_:Tb^3+^. This confirms that ZnO offers a balanced structural environment for Tb^3+^ luminescence. The Judd–Ofelt analysis shows that ZnO is a suitable host for Tb^3+^ ions, providing moderate-to-high local asymmetry, appreciable covalent character, and good structural stability. Among the investigated samples, ZnO:Tb5% exhibits the highest *Ω*_2_, indicating the most favorable local environment for enhancing electric-dipole transitions and green emission from the ^5^D_4_–^7^F_5_ transition.

Based on the optimized Judd–Ofelt intensity parameters obtained for ZnO:Tb^3+^ QDs, further evaluation of the radiative behavior of Tb^3+^ ions can be performed through the calculation of spontaneous emission probabilities for both magnetic-dipole (MD) and electric-dipole (ED) transitions. These parameters are essential for understanding the relative contributions of host-independent magnetic transitions and host-sensitive electric transitions to the overall luminescence performance. In particular, while magnetic-dipole transitions are primarily governed by an intrinsic rare-earth electronic structure and remain nearly unaffected by the surrounding matrix, electric-dipole transitions strongly depend on the local crystal-field environment as reflected by the J–O parameters. Therefore, determining the transition probabilities *A*_MD_ and *A*_ED_ provides deeper insight into the radiative efficiency, branching ratios, and optical suitability of ZnO:Tb^3+^ QDs for photonic applications. The emission probabilities of magnetic-dipole and electric-dipole transitions are calculated using the following equations:^46^13
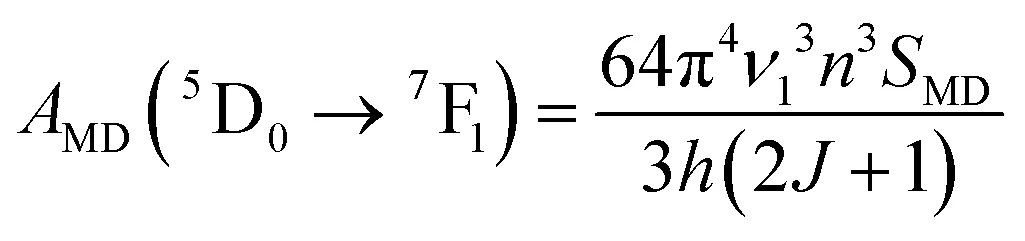
14
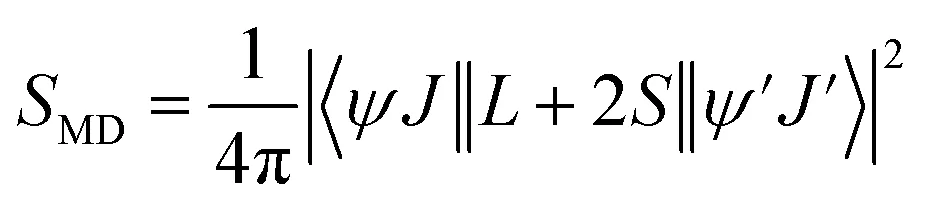
15
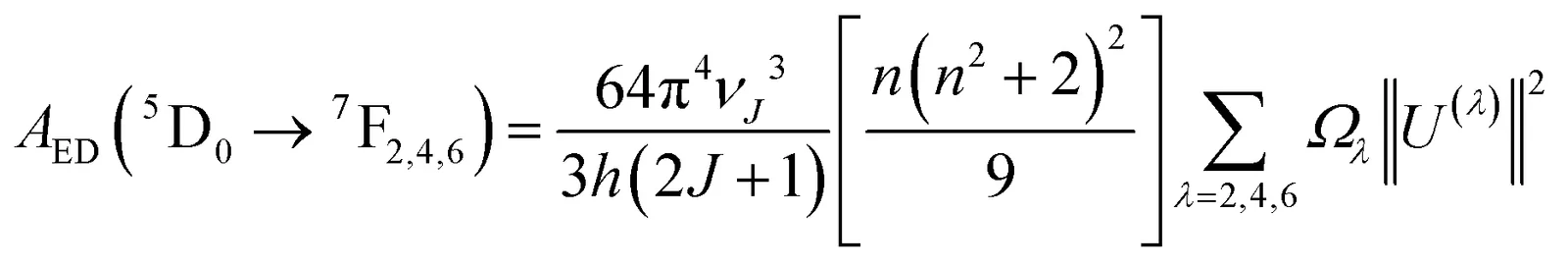
where *ν* denotes the transition wavenumber (or transition energy), *J* represents the total angular momentum quantum number of the emitting excited state, *h* is Planck's constant, and *n* = 2.05 refers to the refractive index of the ZnO host material. *S*_MD_ corresponds to the magnetic-dipole line strength, which is largely determined by the intrinsic electronic configuration of the rare-earth ion and is therefore only weakly influenced by the surrounding host matrix. The terms |〈*ψJ*||*L* + 2*S*||*ψ*′*J*′〉|^2^ are the squared doubly reduced matrix elements of the tensor operators with ranks *λ* = 2, 4, and 6, characterizing the electric-dipole transition probabilities between the relevant 4f electronic states. These matrix elements are fundamental constants for Tb^3+^ ions and are used together with the Judd–Ofelt intensity parameters to evaluate host-dependent radiative transition behavior.

The calculated radiative lifetime (*τ*_cal_) of the emitting ^5^D_4_ level was determined from the total spontaneous emission probability. For Tb^3+^ ions, the total radiative probability (*A*_T_) is obtained by summing the electric-dipole and magnetic-dipole transition probabilities from the ^5^D_4_ level to all lower ^7^F_*J*_ levels:16
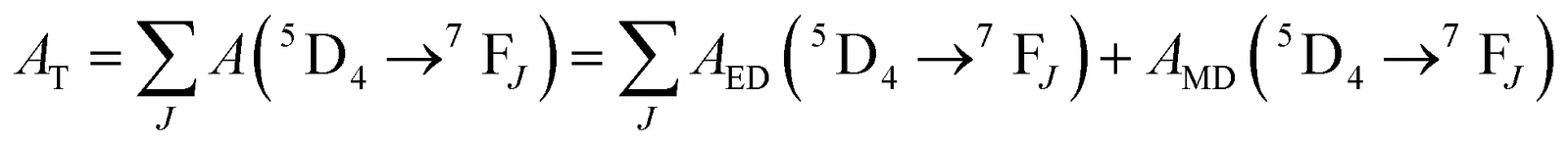


The calculated radiative lifetime is then given by:17
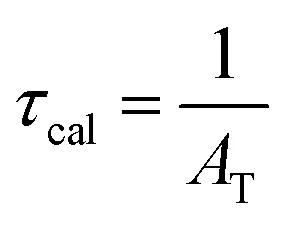
where *A*_T_ is expressed in s^−1^. *τ*_cal_ was calculated and is given in Table 5. This lifetime represents the ideal radiative lifetime of the ^5^D_4_ level when only radiative transitions are considered.

**Table 5:** The radiative parameters of ^5^D_4_ → ^7^F_5_ transition in ZnO:Tb^3+^

Sample	*β*_cal_	*β*_exp_	Δ*λ*_eff_	*τ*_cal_	*σ*_*λ*p_	*σ*_*λ*p_ × Δ*λ*_eff_	*σ*_*λ*p_ × *τ*_cal_
ZnO:Tb0.5%	0.41	0.38	12.72	3.78	2.39	3.04	9.05
ZnO:Tb1%	0.43	0.44	14.06	2.92	2.93	4.12	8.55
ZnO:Tb3%	0.48	0.47	13.83	2.69	2.61	4.99	9.71
ZnO:Tb5%	0.50	0.48	14.49	2.21	4.33	6.28	9.58
ZnO:Tb7%	0.47	0.41	12.79	1.75	5.87	7.51	10.30

The obtained Judd–Ofelt intensity parameters were further employed to evaluate the key radiative characteristics of Tb^3+^ ions, including the calculated branching ratio (*β*_cal_, %), effective emission bandwidth (Δ*λ*_eff_, nm), radiative lifetime (*τ*_cal_, ms), stimulated emission cross-section (*σ*_*λ*p_, 10^−22^ cm^2^), gain bandwidth (*σ*_*λ*p_ × Δ*λ*_eff_, 10^−28^ cm^3^) and optical gain (*σ*_*λ*p_ × *τ*_cal_, 10^−25^ cm^2^ s) for the relevant excited state transitions. These spectroscopic parameters are essential for assessing the emission efficiency, amplification potential, and laser performance of the investigated ZnO host. Detailed computational procedures and the corresponding theoretical framework have been described comprehensively in our previous studies.^40,43,47^

Table 5 summarizes the key radiative parameters of the dominant ^5^D_4_ → ^7^F_5_ green emission transition for ZnO:Tb^3+^ QDs. The *β*_cal_ values increase from 0.41 for ZnO:Tb0.5% to a maximum of 0.50 for ZnO:Tb5%, before slightly decreasing to 0.47 at 7% Tb^3+^ concentration. A similar trend is observed for *β*_exp_ with increasing Tb^3+^ concentration and then declines at higher doping levels. This behavior indicates that moderate Tb^3+^ incorporation enhances radiative transition probability by increasing local asymmetry and electric-dipole strength, while excessive doping introduces concentration quenching, cross-relaxation, or Tb–Tb interactions that reduce radiative efficiency. The effective emission bandwidth (Δ*λ*_eff_) varies between 12.72 and 14.49 nm, indicating relatively broad green emission bands that are advantageous for optical amplification and tunable photonic applications. The widest emission bandwidth is observed for ZnO:Tb5%, suggesting improved spectral coverage and broader gain capability at this concentration. The calculated radiative lifetime (*τ*_cal_) decreases systematically from 3.78 ms at 0.5% Tb^3+^ to 1.75 ms at 7% Tb^3+^. This reduction reflects an increase in the overall radiative transition probability as the Tb^3+^ concentration increases, which is consistent with enhanced electric-dipole transition strength. Shorter radiative lifetimes are generally beneficial for fast optical response and efficient emission processes, although excessively short lifetimes at very high doping may also indicate stronger non-radiative losses.

A particularly important parameter for laser and amplifier applications is the stimulated emission cross-section (*σ*_*λ*p_), which increases significantly from 2.39 × 10^−22^ cm^2^ for ZnO:Tb0.5% to 5.87 × 10^−22^ cm^2^ for ZnO:Tb7%. This continuous increase demonstrates that higher Tb^3+^ concentrations improve the emission strength and optical gain potential of the ZnO host. Likewise, the gain bandwidth parameter (*σ*_*λ*p_ × Δ*λ*_eff_) increases steadily from 3.04 × 10^−28^ cm^3^ to 7.51 × 10^−28^ cm^3^, indicating progressively enhanced amplification bandwidth with increasing Tb^3+^ concentration. The optical gain parameter (*σ*_*λ*p_ × *τ*_cal_) remains relatively high across all samples, ranging from 8.55 to 10.30 × 10^−25^ cm^2^ s, with the highest value observed for ZnO:Tb7%. This suggests that although concentration quenching begins to affect branching ratios at higher doping levels, the material still exhibits strong gain characteristics due to larger stimulated emission cross-sections. The results demonstrated that ZnO:Tb^3+^ QDs exhibit strong green luminescence. Tb^3+^ concentrations in the range of 5–7 mol% provide the most favorable balance among the branching ratio, stimulated emission efficiency, gain bandwidth, and optical amplification performance.

### 3.6. Lifetime of the ^5^D_4_ → ^7^F_5_ transition and quantum efficiency of the ^5^D_4_ level

To further evaluate the practical luminescence performance of ZnO:Tb^3+^ QDs, the decay behavior of the ^5^D_4_ excited state was investigated. The experimental and calculated lifetimes of the ^5^D_4_ → ^7^F_5_ transition provide crucial information about the radiation efficiency, non-radiative losses, and quantum efficiency, which are essential for assessing the suitability of these materials for photonic applications.^18,19,26^

**Fig. 10:**
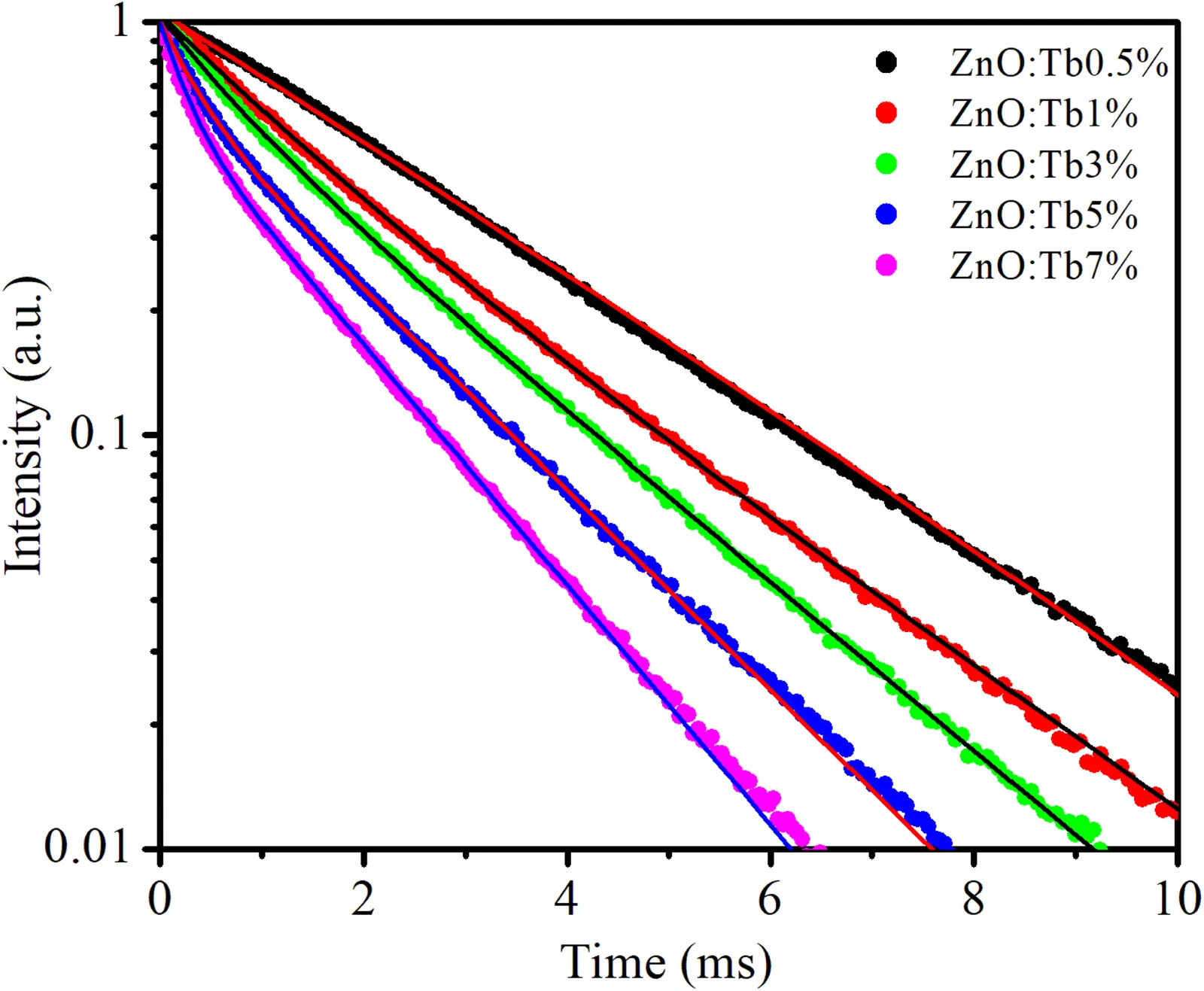
Decay time curves of Tb-doped ZnO QDs measured at 546 nm (^5^D_4_ → ^7^F_5_ transition).

Fig. 10 illustrates the fluorescence decay profiles of Tb^3+^-doped ZnO QDs monitored at 546 nm, corresponding to the ^5^D_4_ → ^7^F_5_ emission transition, under 318 nm excitation. The decay behavior of all investigated samples is well described by a bi-exponential fitting model, indicating the presence of multiple luminescence relaxation channels within the ZnO:Tb^3+^ QDs:^6,13,22^18
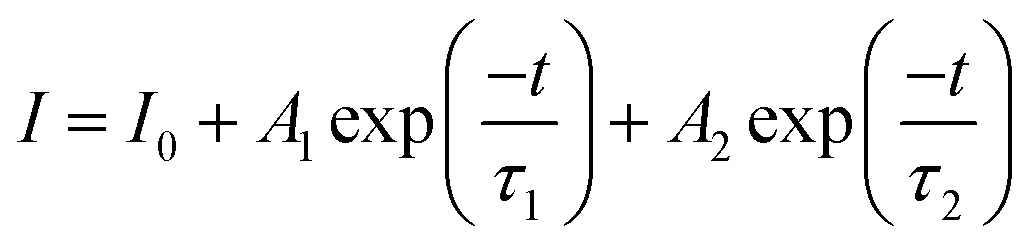
where *I*(*t*) denotes the luminescence intensity at time *t*, *A*_1_ and *A*_2_ are the pre-exponential fitting coefficients, and *τ*_1_ and *τ*_2_ represent the fast and slow decay components, respectively. The shorter lifetime component (*τ*_1_) is typically associated with rapid radiative recombination processes or efficient energy transfer from the ZnO host to Tb^3+^ ions, whereas the longer component (*τ*_2_) is generally attributed to slower relaxation mechanisms, including non-radiative pathways, defect-assisted recombination, or long-lived excited-state decay. The coexistence of these two lifetime components reflects the complex relaxation dynamics of Tb^3+^ ions influenced by both intrinsic electronic transitions and host-related interactions. The average photoluminescence lifetime (*τ*_exp_) for the ^5^D_4_ → ^7^F_5_ transition was subsequently determined using the following formula:^6,13,22^19
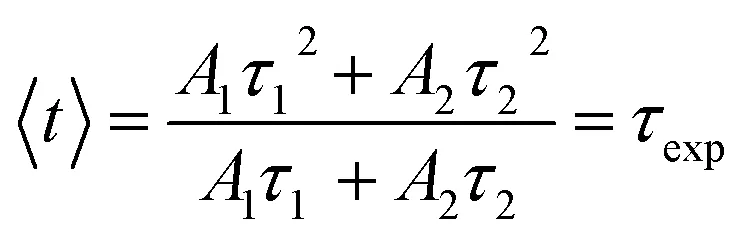


The *τ*_exp_ values of the ^5^D_4_ → ^7^F_5_ transition of ZnO:Tb^3+^ QDs were calculated for all concentrations and are presented in Table 6.

**Table 6:** *τ*_*i*_, *A*_*i*_, and *τ*_exp_ values for ZnO:Tb^3+^ QDs by monitoring 546 nm

Sample	*A*_1_	*τ*_1_ (ms)	*A*_2_	*τ*_2_ (ms)	*τ*_exp_ (ms)
ZnO:Tb0.5%	0.26	0.87	0.74	3.03	2.83
ZnO:Tb1%	0.21	0.65	0.79	2.14	2.03
ZnO:Tb3%	0.23	0.42	0.77	1.74	1.65
ZnO:Tb5%	0.26	0.31	0.74	1.24	1.16
ZnO:Tb7%	0.29	0.13	0.71	0.88	0.84

Table 6 summarizes the bi-exponential fitting parameters (*A*_*i*_, *τ*_*i*_) and average experimental lifetimes (*τ*_exp_) of the ^5^D_4_ → ^7^F_5_ transition for ZnO:Tb^3+^ QDs at different Tb^3+^ concentrations. All samples exhibit two distinct decay components, confirming the coexistence of fast and slow relaxation channels in the luminescence process. The fast decay component (*τ*_1_) decreases significantly from 0.87 ms for ZnO:Tb0.5% to 0.13 ms for ZnO:Tb7%, while the slow component (*τ*_2_) also decreases from 3.03 ms to 0.88 ms over the same concentration range. This systematic reduction in both lifetime components indicates that increasing the Tb^3+^ concentration accelerates the depopulation of the excited ^5^D_4_ level. The dominant contribution of the slow component (*A*_2_ = 0.71–0.79) compared with the fast component (*A*_1_ = 0.21–0.29) suggests that long-lived radiative processes remain the primary luminescence mechanism in ZnO:Tb^3+^ QDs, although their contribution gradually weakens at higher Tb^3+^ concentrations. The increasing *A*_1_ values with Tb^3+^ content imply that faster decay channels, likely associated with non-radiative energy transfer, cross-relaxation, or defect-assisted recombination, become more significant as the dopant concentration increases.

The average experimental lifetime (*τ*_exp_) decreases progressively from 2.83 ms for ZnO:Tb0.5% to 0.84 ms for ZnO:Tb7%. This trend clearly reflects the onset of concentration quenching effects at higher Tb^3+^ concentrations. Several mechanisms may contribute to this behavior: (i) enhanced Tb^3+^–Tb^3+^ energy transfer due to reduced interionic distance, (ii) increased probability of cross-relaxation processes, and (iii) higher defect density or structural disorder introduced by excessive dopant incorporation, which provides additional non-radiative recombination centers. Despite the reduction in lifetime with increasing Tb^3+^ concentration, all samples display millisecond-scale decay times, demonstrating efficient energy confinement within the Tb^3+^ 4f electronic states. Such relatively long-lived green emission is advantageous for optical amplifiers and luminescent devices.

Quantum efficiency and energy transfer probability are crucial parameters for determining how effectively the absorbed excitation energy is converted into radiative emission while also revealing the extent of non-radiative losses and concentration-dependent energy migration processes. The luminescence efficiency (*η*) of the ^5^D_4_ excited state, together with the corresponding energy transfer probability (*W*_ET_), can be quantitatively determined using the following expressions:^43,47,48^20
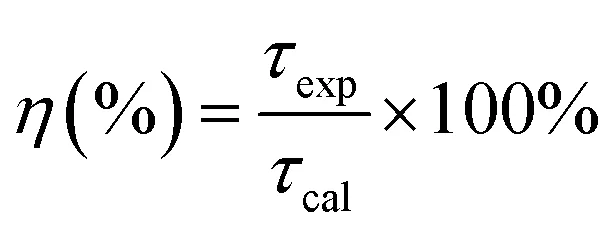
21
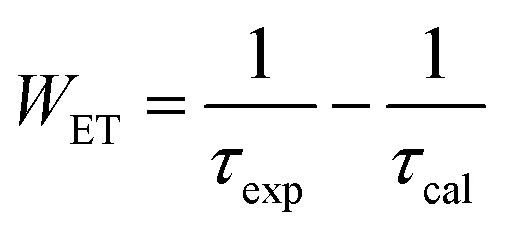
where *τ*_exp_ is the experimentally measured fluorescence lifetime and *τ*_cal_ is the radiative lifetime derived from Judd–Ofelt theoretical calculations. The *η* reflects the fraction of excitation energy that is successfully converted into radiative emission relative to the ideal case in which all excited states decay purely through photon emission. This parameter provides direct insight into the balance between radiative and non-radiative relaxation processes. *W*_ET_ is an important parameter for evaluating how dopant concentration influences luminescence efficiency and for determining the optimal activator concentration for photonic applications.^43,47,48^

Table 7 presents the calculated radiative lifetime (*τ*_cal_), experimental lifetime (*τ*_exp_), quantum efficiency (*η*), and energy transfer probability (*W*_ET_) of the ^5^D_4_ excited state for Tb^3+^ ions in ZnO QDs. A clear systematic trend is observed with increasing Tb^3+^ concentration: both *τ*_cal_ and *τ*_exp_ decrease progressively, while *η* decreases and *W*_ET_ increases significantly. This behavior reflects the increasing influence of Tb^3+^–Tb^3+^ interactions and concentration quenching mechanisms within the ZnO host. The calculated radiative lifetime decreases from 3.78 ms for ZnO:Tb0.5% to 1.75 ms for ZnO:Tb7%, indicating that higher Tb^3+^ concentrations enhance intrinsic radiative transition probabilities due to increased local asymmetry and stronger electric-dipole transition strength, as previously supported by the increase in *Ω*_2_. Simultaneously, the experimental lifetime decreases more sharply from 2.83 ms to 0.84 ms, confirming that non-radiative decay channels become increasingly dominant at elevated dopant concentrations.

**Table 7:** The calculated lifetime (*τ*_cal_, ms), experimental lifetime (*τ*_exp_, ms), quantum efficiency (*η*, %), and energy transfer probability (*W*_ET_, s^−1^) from the ^5^D_4_ level of Tb^3+^ ions in the ZnO host

Sample	*τ*_cal_ (ms)	*τ*_exp_ (ms)	*η* (%)	*W*_ET_
ZnO:Tb0.5%	3.78	2.83	74.87	88.81
ZnO:Tb1%	2.92	2.03	69.52	150.15
ZnO:Tb3%	2.69	1.65	61.34	234.31
ZnO:Tb5%	2.21	1.16	52.49	409.58
ZnO:Tb7%	1.75	0.84	48.00	619.05

The quantum efficiency decreases steadily from 74.87% at 0.5 mol% Tb^3+^ to 48.00% at 7 mol%, demonstrating that although ZnO:Tb^3+^ QDs retain relatively high luminescence efficiency, excessive Tb^3+^ incorporation progressively reduces the fraction of excitation energy converted into useful photon emission. This trend is characteristic of rare-earth-doped luminescent systems, where reduced interionic spacing at higher dopant concentrations promotes energy migration among neighboring activator ions. Similar concentration-dependent reductions in quantum efficiency have been widely reported in other RE^3+^-doped systems, including Eu^3+^-doped CdSe NCs^47^ and Er^3+^-doped ZnO QDs,^48^ where enhanced ion–ion coupling accelerates non-radiative losses.

The probability of energy transfer (*W*_ET_) increased significantly from 88.81 s^−1^ to 619.05 s^−1^ as the concentration of Tb^3+^ increased from 0.5–7%. This nearly eightfold enhancement confirms that energy migration and cross-relaxation processes intensify substantially at higher doping levels. The reduced mean distance Tb^3+^–Tb^3+^ facilitates the transfer of excitation energy between neighboring ions, increasing the probability that excitation energy reaches quenching centers or structural defects before radiation emission occurs. This behavior is consistent with concentration quenching patterns commonly observed in rare earth luminescent hosts, including phosphates, fluorides, and semiconductor QDs.^49,50^

Compared to previously reported Tb^3+^ or RE^3+^ doped hosts, ZnO:Tb^3+^ QDs exhibit relatively high quantum performance at low to medium doping levels, suggesting that ZnO provides an effective host environment that enhances the emission properties of the Tb ion.^51,52^ The relatively high *η* values, especially for ZnO:Tb0.5–3%, indicate superior luminescence retention compared to many doped oxide systems, where photoquenching occurs more rapidly. However, similar to most rare earth hosts, the onset of fluorescence quenching due to ZnO concentration becomes more pronounced when exceeding the optimal concentration range.

### 3.7. Magnetic properties

Besides optimal luminescence properties, the incorporation of Tb^3+^ can also significantly influence the magnetic behavior of ZnO QDs due to the large magnetic moment and the unpaired 4f electrons of the Tb^3+^ ion.^53^ Therefore, studying the magnetic properties of ZnO:Tb^3+^ QDs is essential to understand the interactions between structural, optical, and magnetic functions.

**Fig. 11:**
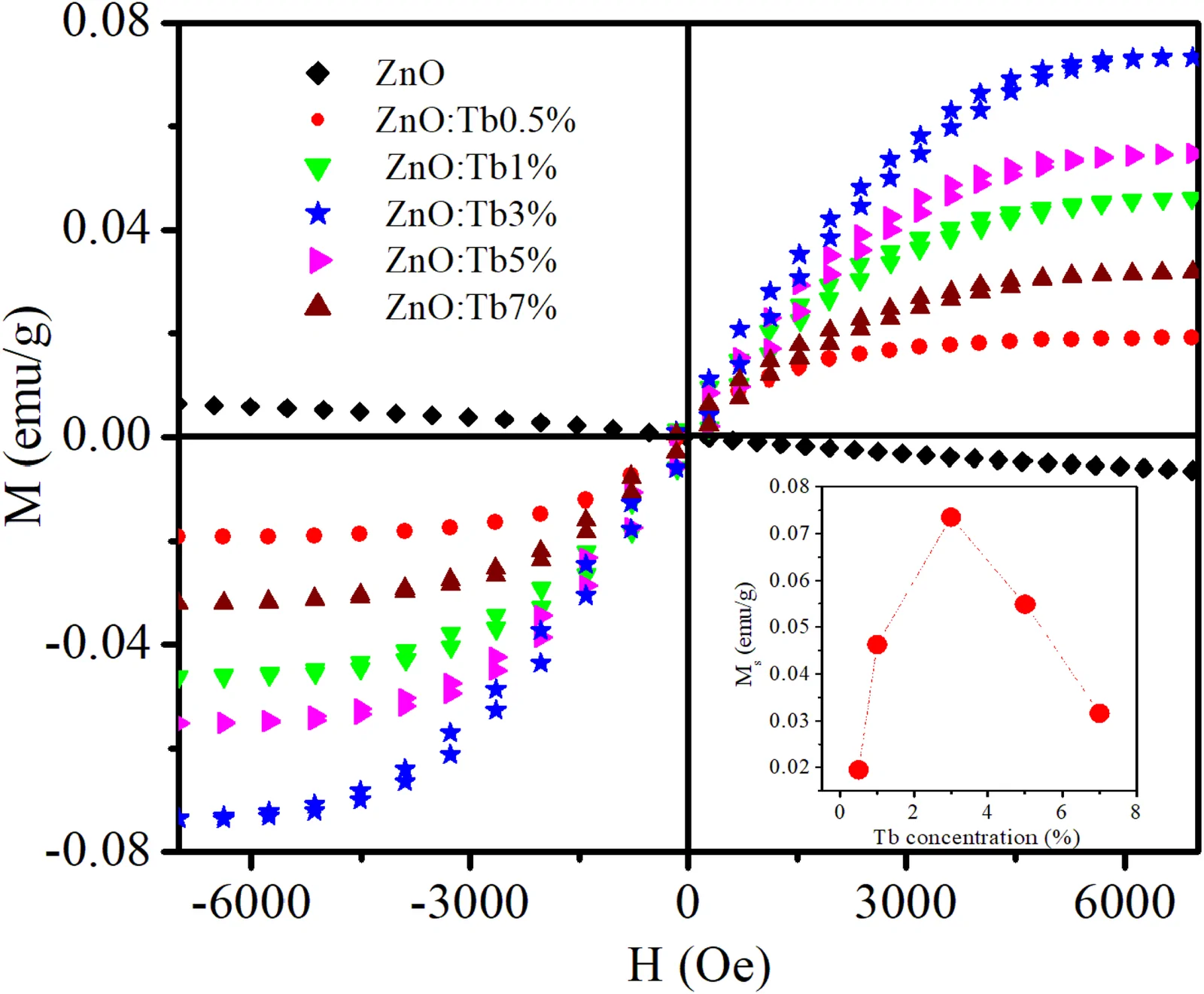
Room temperature M–H plots of ZnO and Tb^3+^-doped ZnO QDs.

Fig. 11 shows the room-temperature magnetization-field (M–H) curves of pristine ZnO and Tb^3+^-doped ZnO QDs, revealing a clear modification of the magnetic response after Tb^3+^ incorporation. The undoped ZnO QDs exhibit a nearly linear M–H dependence with a very small negative/weak magnetic slope and negligible hysteresis, indicating typical diamagnetic behavior. This diamagnetism can be understood from the intrinsic electronic structure of stoichiometric ZnO, in which Zn^2+^ has a fully filled 3d^10^ configuration, and O^2−^ possesses closed-shell electronic states.^54^ Consequently, no unpaired electrons are available to generate permanent magnetic moments, and the magnetization mainly arises from weak field-induced orbital currents that oppose the applied magnetic field. This behavior is consistent with previous reports on undoped ZnO NCs, thin films, and QDs, where pristine ZnO generally shows diamagnetic or very weak paramagnetic responses depending on the concentration of native defects such as oxygen vacancies and zinc interstitials.^55,56^

In contrast, all Tb^3+^-doped ZnO QDs display pronounced S-shaped M–H curves with enhanced magnetization, indicating the emergence of weak ferromagnetic behavior at room temperature. The progressive increase in magnetization upon Tb^3+^ doping confirms that the introduction of Tb^3+^ ions substantially modifies the magnetic interactions within the ZnO lattice. This behavior can be attributed to the magnetic moment of Tb^3+^ ions arising from their partially filled 4f electronic configuration, as well as defect-mediated exchange interactions involving oxygen vacancies or Zn interstitials generated during Tb substitution. Among all investigated samples, ZnO:Tb3% exhibits the highest saturation magnetization (*M*_s_), reaching approximately 0.073 emu g^−1^, as shown in the inset of Fig. 11. This suggests that the 3 mol% Tb^3+^ concentration provides the optimal balance between effective magnetic ion incorporation and sufficient interionic spacing to maximize ferromagnetic exchange interactions. At lower concentrations (0.5–1%), the magnetic response is weaker due to insufficient magnetic ion coupling, while at higher concentrations (5–7%), the saturation magnetization decreases. This reduction may result from increased Tb–Tb antiferromagnetic interactions, clustering effects, or concentration-induced disorder that partially suppresses long-range ferromagnetic ordering.^57,58^ The non-monotonic dependence of magnetization on Tb^3+^ concentration strongly suggests that the magnetic properties of ZnO:Tb^3+^ QDs are governed by an interplay among dopant concentration, structural defects, and exchange mechanisms. Similar behavior has been reported in other rare-earth-doped diluted magnetic semiconductors, where optimal dopant concentrations maximize defect-mediated ferromagnetism, while excessive doping leads to magnetic dilution or competing antiferromagnetic coupling.^59,60^

## 4. Conclusion

Tb^3+^-doped ZnO QDs were synthesized to tune both optical and magnetic properties of ZnO. The samples with different Tb^3+^ concentrations were prepared by a wet-chemical precipitation method. Their structure, composition, optical emission, lifetime, Judd–Ofelt parameters, and magnetic behavior were investigated. XRD and Rietveld refinement confirmed the formation of hexagonal wurtzite ZnO. No secondary phase was detected. The slight lattice expansion and increased microstrain indicate the successful incorporation of Tb^3+^ ions into the ZnO lattice. XPS and EDX mapping further confirmed the presence and uniform distribution of Tb in the samples. UV-vis analysis showed a red shift of the absorption edge and a decrease in the band gap with increasing Tb^3+^ concentration. The PL spectra exhibited the characteristic green emission of Tb^3+^, mainly from the ^5^D_4_ → ^7^F_5_ transition at about 546 nm. The emission intensity reached its maximum at 5 mol% Tb^3+^ and then decreased because of concentration quenching. Judd–Ofelt analysis showed that ZnO:Tb5% has favorable radiative parameters for green emission. Lifetime results confirmed stronger non-radiative relaxation at higher Tb^3+^ concentrations. Magnetic measurements showed that Tb^3+^ doping changed diamagnetic ZnO into weak ferromagnetic-like materials at room temperature. The highest saturation magnetization was obtained for ZnO:Tb3%. These results show that Tb^3+^ doping is an effective way to develop multifunctional ZnO quantum dots for photonic, magneto-optical, and spintronic applications.

## Author contributions

N. T. Hien: writing – original draft, methodology, investigation. N. T. M. Thuy, V. H. Yen: methodology, investigation, experiment. N. T. Luyen, L. V. Hoang: data curation, conceptualization. N. T. K. Van: investigation, formal analysis. L. X. Hung, T. Ngoc: experiment, methodology, investigation. N. X. Ca: writing – review & editing, methodology, investigation.

## Conflicts of interest

The authors declare that they have no known competing financial interests or personal relationships that could have appeared to influence the work reported in this paper.

## Data Availability

The data that support the findings of this study are available from the corresponding author upon reasonable request.
